# SUMOylation of TP53INP1 is involved in miR-30a-5p-regulated heart senescence

**DOI:** 10.1038/s12276-024-01347-3

**Published:** 2024-11-07

**Authors:** Yi-Xiang Hong, Chan Wu, Jing-Zhou Li, Fei Song, Yu Hu, Yue Han, Yi-Jie Mao, Wei-Yin Wu, Yan Wang, Gang Li

**Affiliations:** 1 Xiamen Cardiovascular Hospital of Xiamen University, School of Medicine, Xiamen University, Xiamen, 361000 Fujian China; 2 Xiamen Key Laboratory of Cardiovascular Diseases, Xiamen, 361000 Fujian China

**Keywords:** Senescence, Cardiac hypertrophy

## Abstract

Heart senescence is critical for cardiac function. This study aimed to characterize the role and mechanism of action of miR-30a-5p in cardiac senescence. miR-30a-5p was downregulated in aged mouse hearts and neonatal rat cardiomyocytes (NRCMs). In vivo, using a combination of echocardiography and different molecular biological approaches, we investigated the role of miR-30a-5p knockout or overexpression in natural- or D-galactose-induced heart aging in mice. In vitro, using RNA sequencing and a series of molecular biology methods, the mechanism by which miR-30a-5p regulates cardiac senescence was explored in cardiomyocytes. miR-30a-5p knockout mice showed aggravated natural- or D-galactose-induced heart aging compared to wild-type littermate mice, with significantly decreased heart function, an increased number of γH2AX-positive cells, reduced telomere length, and upregulated p21 and p53 expression. Cardiac-specific knockdown of miR-30a-5p using adeno-associated virus 9 in D-galactose-induced senescent wild-type mice resulted in effects similar to those observed in knockout mice. Notably, the overexpression of miR-30a-5p in wild-type murine hearts alleviated D-galactose-induced heart senescence by improving heart function, increasing telomere length, decreasing the number of γH2AX-positive cells, and downregulating p53 and p21 expression. This was confirmed in D-galactose-treated or naturally aged NRCMs. Mechanistically, TP53INP1 was identified as a target of miR-30a-5p by mediating the SUMOylation of TP53INP1 and its translocation from the cytoplasm to the nucleus to interact with p53. Furthermore, this study demonstrated that cardiac-specific TP53INP1 deficiency ameliorates miR-30a-5p knockout-aggravated cardiac dysfunction and heart senescence. This study identified miR-30a-5p as a crucial modulator of heart senescence and revealed that the miR-30a-5p–TP53INP1–p53 axis is essential for heart and cardiomyocyte aging.

## miR-30a-5p modulates heart aging through TP53INP1 pathway

As we get older, our hearts change, leading to heart diseases, a major cause of death worldwide. A recent study looked at the role of miR-30a-5p, a tiny RNA molecule, in heart aging. Researchers found that lower levels of miR-30a-5p in the heart contribute to aging. They used genetic engineering to change miR-30a-5p levels in mice and saw how it affected heart health and aging. Lower levels of miR-30a-5p led to worse heart function, shorter telomeres, and more signs of aging. But, increasing miR-30a-5p levels protected against these effects. The study identified a protein, TP53INP1, that links miR-30a-5p to heart cell aging. The findings show that miR-30a-5p is crucial in heart aging by controlling heart cell health and lifespan. By targeting miR-30a-5p, new treatments could be made to slow heart aging and prevent related diseases. This summary was initially drafted using artificial intelligence, then revised and fact-checked by the author.

## Introduction

During natural aging, physiological remodeling of the heart is a critical component of the development of cardiovascular disease (CVD) and heart failure (HF)^1,2^. Age-related heart remodeling in the left ventricle causes structural and functional changes in the heart that are associated with high morbidity and mortality worldwide, which are induced not only by prolonged exposure to cardiovascular risks but also by intrinsic cardiac aging^3^. Intrinsic cardiac aging is defined by progressive left ventricular hypertrophy and atrial fibrillation, slow age-dependent degeneration and a decline in diastolic function, which reduces cardiac functional reserve, predisposes the heart to stress and contributes to increased cardiovascular mortality in elderly individuals^4,5^. Independent of traditional risk factors, aging is a significant risk factor for HF development^6^. In older individuals, CVD risk and mortality are increased, as the accumulation of extracellular matrix in the myocardium and collagen deposition in the vascular media lead to a progressive decline in cardiac function^7,8^.

Cellular senescence is defined as the permanent cessation of cell proliferation^9^. Instead of being removed from the body, senescent cells accumulate over a lifetime, which is considered both a hallmark and a contributor to aging^10^. Senescent cells, e.g., cardiomyocytes, are characterized by several traits, including increased levels of p21, p53, and p16^11,12^ and prominent expression of β-galactosidase (β-gal)^13^. Telomere shortening is also considered an essential hallmark of aging^14^. Unfortunately, no effective treatment to prevent heart aging from causing heart dysfunction is known. Therefore, novel therapeutic strategies capable of limiting cardiovascular events in aging patients are urgently needed.

miR-30a-5p has been reported to be related to kidney injury, cancer, autoimmune diseases, Alzheimer’s disease, and other diseases^15–17^. Our group demonstrated the therapeutic potential of miR-30a-5p in atherosclerosis through the regulation of macrophage polarization and lipid metabolism^18^. Several studies have shown that miR-30a is involved in CVD, such as an association between circulating miR-30a and acute myocardial infarction (MI) or HF^19,20^, a role in Ang-(1-7)-inhibited mitochondrial fission in high-glucose–treated podocytes^21^, and it is related to attenuated mitochondrial impairment and myocardial apoptosis caused by epigallocatechin gallate pretreatment^22^. Moreover, miR-30a is modulated by aging and is related to keratinocyte differentiation^23^. However, whether miR-30a-5p exerts cardioprotective effects on cardiac aging has yet to be explored. In the present study, we had two main goals: 1) to investigate the impact of miR-30a-5p overexpression or deletion on naturally or D-galactose (D-gal)-induced aged hearts in vivo and in vitro and 2) to explore the molecular signaling pathways involved in the regulation of heart aging and cardiac function by miR-30a-5p.

## Methods

### Reagents and antibodies

The Senescent Cells IHC Kit (Cat# CS0030) and D-gal (Cat# G0750-25G) were purchased from Sigma‒Aldrich (St. Louis, MO, USA). RIPA lysis buffer (containing 50 mM Tris, 150 mM NaCl, 1% Triton X-100, 1% sodium deoxycholate, and 0.1% SDS; Cat# R0010), phosphatase inhibitor (Cat# P1260), protease inhibitor (Cat# P6730), BCA kit (Cat# PC0020), SDS‒PAGE 5× loading buffer (Cat# P1040), Western blotting substrate (Cat# PE0010), and 4’,6-diamidino-2-phenylindole (DAPI; Cat# C0060) were obtained from Solarbio (Beijing, China). The miRNA-related kits, including the All-in-One™ miRNA First-Strand cDNA Synthesis Kit (Cat# QP114), the qPCR primer against mature miRNA mmu-miR-30a-5p (Cat# MmiRQP0391), the Mouse snRNA U6 qPCR Primer (Cat# MmiRQP9002), and the miR-30a-5p FISH SA-Biotin Kit (Cat# F32102/50), were provided by GeneCopoeia (Rockville, MD, USA). The High-Capacity cDNA Archive Kit (Cat# 4374967), TRIzol reagent (Cat# 15596018), Lipofectamine^TM^ RNAiMAX (Cat# 13778150), and Accutase Enzyme Cell Detachment Medium (Cat# 00-4555-56) were purchased from Thermo Fisher Scientific (Carlsbad, CA, USA). AAV9-cTnT-mmu-miR-30a-5p-overexpression-LUC (1.5 ×10^12^ vg/mL, Lot# 34082017), AAV9-cTnT-NC-overexpression-LUC (1.7 ×10^12^ vg/mL, Lot# 34082016), AAV9-cTnT-mmu-miR-30a-5p-sponge-LUC (1.5 ×10^12^ vg/mL, Lot# 34082019), and AAV9-cTnT-LUC-NC (1.8 ×10^12^ vg/mL, Lot# 34082018) were purchased from HANBIO (Shanghai, China). AAV9-cTnT-TP53INP1-RNAi (2.0 ×10^12^ vg/mL, Cat# GIDV0326887) and AAV9-cTnT-NC (2.0 ×10^12^ vg/mL, Cat# GIDV0326888) were obtained from Genechem (Shanghai, China). ABScript III RT Master Mix for qPCR with gDNA remover (Cat# RK20429) and 2X Universal SYBR Green Fast qPCR Mix (Cat# RK21203) were obtained from ABclonal (Wuhan, China). miRNA-related reagents, such as mus-miR-30a-5p-mimics (sense: 5’-UGUAAACAUCCUCGACUGGAAG-3’, antisense: 5’-UCCAGUCGAGGAUGUUUACAUU-3’), negative control (sense: 5’-UUCUCCGAACGUGUCACGUTT-3’, antisense: 5’-ACGUGACACGUUCGGAGAATT-3’), mus-mir-30a-5p-inhibitor (5’-CUUCCAGUCGAGGAUGUUUACA-3’), and inhibitor control (5’-CAGUACUUUUGUGUAGUACAA-3’), were from GenePharma (Guangzhou, China). *Tp53inp1*-siRNA (Cat# A10001) and control siRNA (Cat# A10002) were also provided by GenePharma. The following primers including the rat primers *Senp1* (F: GGACCAGCTTGCACTTTCTG, R: CTTGCTGACAAAGAATCCTGAG), *Sumo1* (F: GAAGCTCAAAGAATCGTACTGTC, R: TCTTCTTCCTCCATTCCCAGTTC), *Senp6* (F: ACACAAGCTACGGCGTTATG, R: TTTCGGTCCTGATTGCTCTG), *Pias4* (F: AGCTGGTGGAGGCCAAAAAC, R: CCGCTCTTGCTACGACCTAC), *Cbx4* (F: TGCCTTCCAGAACAGGGAAAG, R: GCCCAGTCAGGACATTGGAG), *Tp53inp1* (F: TATTTCCGCCTGTACCTTGGG, R: TCGTGGCTGGAAGAAGTAGTG), *Ddit4* (F: TCTTGTCCGCAATCTTCGCT, R: GGAGGACGAGAAACGATCCC), and *Gapdh* (F: GCCTGGAGAAACCTGCCAAG, R: GGTGGAAGAATGGGAGTTGC); and mouse primers *Senp1* (F: ACTCCAAGTGCGACGAAGAG, R: ACAGAATCCGAACCGGAACC), *Sumo1* (F: GGAAGTGACGCAAGACGTAGA, R: TCAGACATGGTGACGTGGAT), *Senp6* (F: TGTAAGGCGGAGCAAGACTG, R: TCTGAGCCGCTGTTCTGATG), *Pias4* (F: GTGTGGAACCTAAGAGGCCC, R: CTCTTGCCATAGTTGCCCCA), *Cbx4* (F: TTCTCCAGGAACTGCGACAC, R: CCCTCAACTTCGGACCTTCC), IL‐1β (F: TGGACCTTCCAGGATGAGGACA, R: GTTCATCTCGGAGCCTGTAGTG), IL-6 (F: TCCAGTTGCCTTCTTGGGACTGA, R: TAAGCCTCCGACTTGTGAAGTGGT), *Gapdh* (F: TCTACATGTTCCAGTATGACTC, R: ACTCCACGACATACTCAGCACC), telomere primer (F: GGTTTTTGAGGGTGAGGGTGAGGGTGAGGGTGAGGGT, R: TCCCGACTATCCCTATCCCTATCCCTATCCCTATCCCTA), and 36B4 (F: CACACTCCATCATCAATGGGTACAA, R: CAGTAAGTGGGAAGGTGTACTCA) were purchased from Brogene Biotechnology (Xiamen, China). Wheat germ agglutinin (WGA, Cat# L4895) was purchased from Sigma‒Aldrich (St. Louis, MO, USA). The Picrosirius Red Staining Kit (Cat# Ab245887) was purchased from Abcam (Cambridge, UK). Human SUMO1 (MSDQEAKPSTEDLGDKKEGEYIKLKVIGQDSSEIHFKVKMTTHLKKLKESYCQRQGVPMNSLRFLFEGQRIADNHTPKELGMEEEDVIEVYQEQTGGHSTV), human wild-type TP53INP1 (MFQRLNKMFVGEVSSSSNQEPEFNEKEDDEWILVDFIDTCTGFSAEEEEEEEDISEESPTEHPSVFSCLPASLECLADTSDSCFLQFESCPMEESWFITPPPCFTAGGLTTIKVETSPMENLLIEHPSMSVYAVHNSCPGLSEATRGTDELHSPSSPRVEAQNEMGQHIHCYVAALAAHTTFLEQPKSFRPSQWIKEHSERQPLNRNSLRRQNLTRDCHPRQVKHNGWVVHQPCPRQYNY), and TP53INP1^K113R^ mutants (MFQRLNKMFVGEVSSSSNQEPEFNEKEDDEWILVDFIDTCTGFSAEEEEEEEDISEESPTEHPSVFSCLPASLECLADTSDSCFLQFESCPMEESWFITPPPCFTAGGLTTIRVETSPMENLLIEHPSMSVYAVHNSCPGLSEATRGTDELHSPSSPRVEAQNEMGQHIHCYVAALAAHTTFLEQPKSFRPSQWIKEHSERQPLNRNSLRRQNLTRDCHPRQVKHNGWVVHQPCPRQYNY) were subcloned and inserted into the pEGFP-C1 vector (Clontech, Mountain View, CA, USA) by SANGON Biotech (Shanghai, China).

The primary antibodies used for western blotting were mouse monoclonal anti-p53 (Cat# Ab26, Abcam, Cambridge, UK), rabbit polyclonal anti-p21 (Cat# 8248-1-AP) and mouse monoclonal anti-TERF2 (Cat# 66893-1-Ig, both ProteinTech, Wuhan, China), rabbit polyclonal anti-TP53INP1 (Cat# OM204116, OmniMabs, Alhambra, CA, USA), mouse monoclonal anti-SUMO1 (Cat# sc-5308, Santa Cruz, Dallas, Texas, USA), rabbit polyclonal anti-TERT (Cat# DF7129), rabbit polyclonal anti-DDIT4 (Cat# 10638-1-AP, Proteintech, Wuhan, China), and rabbit polyclonal anti-SENP1 (Cat# AF0275, both Affinity Bioscience, Nanjing, China). The rabbit monoclonal anti-gamma γH2A. The X antibody (Cat# Ab81299) used for immunofluorescence staining was obtained from Abcam. The mouse monoclonal β-Gal antibody (Cat# 2372S) was obtained from Cell Signaling Technology.

### Animals

All animal study procedures were performed in accordance with the guidelines of Directive 2010/63/EU of the European Parliament on the protection of animals used for scientific purposes. All of the animal experiments complied with the guidelines of the Institutional Animal Care and Use Committee of Xiamen University and were authorized by the Ethics Committee of Xiamen University (approval no. XMULAC20190120). WT male C57BL/6 mice were purchased from GemPharmatech (Nanjing, China). miR-30a-5p KO mice were obtained from the Laboratory Animal Center of Xiamen University. All of the mice were maintained in the specific pathogen-free-grade Laboratory of the Animal Center of Xiamen University. The mice were provided food and water ad libitum under a 12-h light/dark cycle from 06:00–18:00. The mice were anesthetized via isoflurane inhalation (induction, 3%; maintenance, 1.5%; Cat# R510-22; RWD, Shenzhen, China) using a small animal anesthesia system (Cat# R550, RWD) and were euthanized via intoxication with 100% carbon dioxide.

### Generation of miR-30a-5p KO mice

miR-30a-5p KO mice were generated by the Laboratory Animal Center of Xiamen University. CRISPR/Cas9 technology was used to produce miR-30a-5p-KO mice. A total of 100 ng/μL Cas9 and 50 ng/μL gRNA were injected into fertilized eggs of C57BL/6J mice. The eggs were subsequently transplanted into ICR pseudopregnant female mice. The Cas9 primer sequences were F: 5’-CACCGACTGAGCTCCTTAAG-3’ and R: 5’-TAGTCAAGCTTCCATGGCTCGA-3’. The F0 generation of heterozygous mice (miR-30a-5p^+/-^) was screened. These mice were crossed repeatedly until miR-30a-5p pure-hybrid mice (miR-30a-5p^-/-^) were generated. The genotype of miR-30a-5p KO was validated using PCR with the following primers: F: 5’-AGCTTCCCTACTTTGGTGTTT-3’ and R: 5’-TGGTGTGTGTGAATTGACCT-3’. All investigations used WT mice and their miR-30a-5p KO littermates.

### D-galactose treatment to induce the aging model

D-gal was used to generate an animal aging model, as previously described^24,25^. D-gal was dissolved in phosphate-buffered saline (PBS) at a concentration of 100 mg/mL. A dose of 150 mg/kg was randomly injected subcutaneously into WT or KO mice daily for approximately 90 days. The mice were subsequently anesthetized via isoflurane inhalation for echocardiography. The mice were then euthanized by intoxication with 100% CO_2_, and the blood and heart were collected for subsequent analyses.

### Animal treatments

To investigate the effects of miR-30a-5p deficiency on natural heart aging, male KO mice and age-matched littermate WT male mice (C57BL/6J) were randomly divided into 2- or 18-month maintenance groups. The mice were subsequently subjected to echocardiography. After euthanasia, blood and heart tissue samples were collected for immunofluorescence, qRT‒PCR, and western blotting.

To determine the role of miR-30a-5p KO in D-gal-induced heart senescence, 10-week-old male WT and age-matched male littermate KO mice were randomly injected with D-gal (150 mg/kg) for 90 days. The mice were subsequently subjected to echocardiography and sacrificed for sample collection. The heart samples were embedded in Sakura Tissue-Tek O.C.T. compound (Sakura, Tokyo, Japan) or stored at −80 °C to determine their histology and protein expression.

To explore the effects of cardiac-specific deficiency of miR-30a-5p on D-gal-induced heart senescence, 10-week-old male WT mice were randomly injected with 100 µL of AAV9-miR-30a-5p sponge (AAV9-cTnT-mmu-mir-30a-5p-sponge-LUC, Lot# 34082019, 1.5 ×10^12^ vg/mL, HANBIO) or AAV9-control sponge (AAV9-cTnT-LUC-NC, Lot# 34082019, 1.8 ×10^12^ vg/mL, HANBIO) via the tail vein. After AAV9 injection, D-gal was injected subcutaneously into the mice from days 0 to 89. On day 90, the mice were subjected to echocardiography and subsequently sacrificed for heart tissue collection.

The effect of cardiac-specific overexpression of miR-30a-5p on D-gal-induced heart senescence was determined by randomly injecting AAV9-miR-30a-5p (100 µL, AAV9-cTnT-mmu-mir-30a-5p-Null-LUC, 1.5 ×10^12^ vg/mL, Lot# 34082016, HANBIO) or AAV9-control (100 µL, AAV9-cTnT-mmu-NC-Null-LUC, 1.7 ×10^12^ vg/mL, Lot# 34082017, HANBIO) into the tail veins of WT mice. Six days after AAV9 injection, vehicle or D-gal was randomly subcutaneously injected into the mice from days 6 to 89 to induce senescence. The mice were subsequently subjected to echocardiography and euthanized for heart tissue collection.

To explore the role of TP53INP1 in D-gal-induced senescence in WT and KO mice, cardiac-specific knockdown was induced by randomly grouping and injecting AAV9-TP53INP1 (AAV9-cTnT-TP53INP1-RNAi, 100 µL, 2 ×10^12^ vg/mL) or AAV9-control (AAV9-cTnT-NC, 100 µL, 2 ×10^12^ vg/mL) via the tail vein. Afterward, D-gal was injected into the four groups for 90 days to induce heart senescence. Three months later, the cardiac function of the mice was assessed via echocardiography, and the mice were euthanized. Heart tissues were collected for histological, immunofluorescence, qPCR, and western blot analyses.

### RNA fluorescence in situ hybridization

RNA-FISH was conducted to determine the expression of miR-30a-5p in the hearts of young and aging mice using the miR-30a-5p FISH SA-Biotin Kit (Cat# F32102/50; GeneCopoeia, Rockville, MD, USA). The heart samples were embedded in Sakura Tissue-Tek O.C.T. and cut into 6 μm sections. Following the manufacturer’s instructions, the sections were rehydrated in citrate buffer, digested with proteinase K, denatured at 78 °C, and incubated in the probe working solution. Next, the sections were rinsed with posthybridization aqueous solution, and the nuclear DNA was stained with DAPI. A Leica SP5 II confocal microscope was used to capture the images. ImageJ software (National Institutes of Health, MD, USA) was used to analyze these images.

### Immunofluorescence staining

The heart tissues from the mice were harvested and embedded in Sakura Tissue-Tek O.C.T. and stored in a −80 °C freezer. Then, tissue cross-sections of 6 μm thickness were cut and fixed in precooled acetone for 10 min. Afterward, the sections were permeated with 0.1% Triton X-100 in Tris-buffered saline with Tween 20 (TBST) for 15 min, incubated in 10% secondary-derived serum for 60 min, and then incubated with the primary antibodies anti-γH2AX, anti-TP53INP1, anti-β-gal, or anti-p53 (diluted at 1:100) at 4 °C overnight. After rinsing with TBST, the sections were incubated with the corresponding secondary antibodies conjugated to different fluorescent molecules for 1 h. Nuclear DNA was counterstained with DAPI. The sections were imaged using a Leica SP5 II confocal fluorescence microscope (Leica, Germany).

### Echocardiography

The mice were anesthetized via isoflurane inhalation (induction, 3%; maintenance, 1.5%; Cat# R510-22; RWD, Shenzhen, China) using a small animal anesthesia system (Cat# R550, RWD). Transthoracic echocardiography was performed by blinded investigators using a Vevo 2100 animal ultrasound imaging system (VisualSonics, Toronto, Canada) with a 30 MHz center frequency transducer. Systolic and diastolic LV volumes were calculated using the area‒length method, and the LVEF and LVFS were derived.

### Isolation of neonatal rat cardiomyocytes

The hearts of newborn 1–3-day-old rats were removed and washed with precooled PBS. The hearts were then cut into pieces and stirred overnight at 4 °C. After incubation with 1.5 mg/mL collagenase II for 30 min at 37 °C on a shaker, individual myocardial cells were separated. The cells were then cultured in dishes for 1–3 h. After the fibroblasts and endothelial cells had adhered to the dishes, the upper layer of the myocardial cell suspension (1.5 ×10^5^ cells/cm^2^) was spread onto culture plates pretreated with 0.1% collagen solution. After 24 h, the cardiomyocytes had attached to the plates and begun to beat. The medium was changed to prewarmed maintenance medium (78% DMEM, 17% M-199 medium, 4% horse serum, and 1% penicillin‒streptomycin solution).

### NRCM transfection and treatment

After isolation, the NRCMs were transferred to 6-well plates and incubated for 12 h. The cells were then cotransfected with Lipofectamine^TM^ RNAiMAX Transfection Reagent (8 μL) and the miR-30a-5p inhibitor (final concentration of 150 nM), inhibitor control (final concentration of 150 nM), miR-30a-5p mimics (final concentration of 100 nM), negative control (final concentration of 100 nM), *Tp53inp1*-siRNA (final concentration of 100 nM), or control siRNA (final concentration of 100 nM) in 1 mL of Opti-MEM (Life Technology). Six to eight hours after transfection, the medium was replaced with DMEM (with 10% FBS). For D-gal treatment (final concentration of 30 mg/mL), 48 h after transfection, the cells were incubated with D-gal or vehicle for an additional 72 h. For natural aging, the cells were maintained in DMEM (with 10% FBS) for a total of 5 or 10 days after isolation. The medium was changed every two days. The cells were then prepared for subsequent detection, such as SA-β-gal staining, western blotting, and qRT‒PCR.

### Senescence-associated β-galactosidase staining

SA-β-gal staining was conducted to detect aging cells using the Senescent Cells IHC Kit. Following the manufacturer’s instructions, the cells were fixed with 4% paraformaldehyde for 5 min after treatment or transfection. The staining solutions were prepared. After being washed with PBS three times, the cells were stained with the staining solution and incubated at 37 °C in a CO_2_-free incubator overnight. After washing, images were captured using a Leica microscope. The number of positive cells in the images was analyzed via ImageJ.

### Quantitative real-time polymerase chain reaction

After treatment and collection, mouse heart tissues and NRCMs were homogenized using TRIzol reagent. A total of 200 µL of chloroform was added, mixed, and centrifuged at 13,200 × *g* for 15 min. The upper layer was collected, combined with 75% ethanol, and centrifuged at 13,200 × *g* for 10 min. The RNA was collected, and its concentration was measured. cDNA was produced using a miRNA reverse transcription kit (All-in-One™ miRNA First-Strand cDNA Synthesis Kit) or a High-Capacity cDNA Archive Kit. To detect the expression of miR-30a-5p in mice or NRCMs, we applied a miR-30a-5p qPCR primer kit (qPCR primer against mature miRNA mmu-miR-30a-5p Kit) and an internal reference control snRNA U6 primer Kit (Mouse snRNA U6 qPCR Primer) and mixed them with cDNA and ABScript III RT Master Mix from the qPCR Kit. To detect the expression of *Senp1*, *Sumo1*, *Senp6*, *Pias4*, *Cbx4*, *Tp53inp1*, and *Ddit4* in rat NRCMs, we mixed the rat primers with ABScript III RT Master Mix from the qPCR Kit. Rat *Gapdh* was used as the internal reference control. To detect the expression of *Senp1*, *Sumo1*, *Senp6*, *Pias4*, and *Cbx4* in WT and KO mice, these mouse primers were mixed with cDNA and ABScript III RT Master Mix from the qPCR Kit, and mouse *Gapdh* was used as the internal reference control. To determine the telomere length in the mice, the telomere primers were mixed with cDNA and the ABScript III RT Master Mix from the qPCR Kit and Mouse 36B4 were used as the internal reference. After mixing, the expression levels were detected via a qPCR system (CFX96 Touch Real-Time PCR Detection System; Bio-Rad, Hercules, CA, USA). The rat telomere length primer and rat internal reference 36B4 were used to measure the telomere length in NRCMs with the same qRT‒PCR kit.

### Immunoprecipitation assays

For the immunoprecipitation assays, heart samples from WT and KO mice were washed with PBS and lysed in RIPA lysis buffer. The samples were centrifuged at 13,200 × *g* to obtain proteins and then incubated with anti-p53 antibody or anti-TP53INP1 antibody at 4 °C overnight. The protein‒antibody conjugates were then incubated with protein A/G agarose beads (Santa Cruz) at 4 °C for 3–5 h. After being washed four times with lysis buffer, the protein was boiled in SDS‒PAGE sample buffer and analyzed via western blotting with anti-p53, anti-TP53INP1, or anti-SUMO1 antibodies.

### Western blotting assays

Heart tissue or NRCM samples were homogenized in RIPA lysis buffer containing 1% phosphatase inhibitor and 1% protease inhibitor for 30 min. The protein concentration was quantified using a BCA kit. After the addition of 5× loading buffer (1/5 volume) for SDS‒PAGE, the samples were denatured for 5 min at 95 °C. SDS–PAGE was performed to separate proteins of different molecular weights, and the samples were then transferred onto a PVDF membrane. The membranes were blocked with 5% skim milk for 1 h. After that, the membranes were incubated with primary antibodies against p53, p21, TERF2, TP53INP1, SUMO1, TERT, DDIT4, and SENP1 overnight at 4 °C. After being washed three times, the membranes were incubated with secondary antibodies for 1 h. Finally, the stained protein bands were visualized with enhanced chemiluminescence western blotting Substrate and a ChemiDoc MP chemiluminescence imaging system (Bio-Rad). The gray values of the bands were calculated via ImageJ (National Institutes of Health, MD, USA).

### Statistical analysis

All of the data were statistically analyzed by blinded investigators using GraphPad Prism 9 statistical software. The data are expressed as the means ± standard errors (SEMs). Student’s unpaired t test was used to compare two groups. One-way or two-way analysis of variance (ANOVA) with multiple comparison tests was used to compare three or more groups. Statistical significance was set at *P* < 0.05.

## Results

### Heart senescence decreases miR-30a-5p expression

**Fig. 1:**
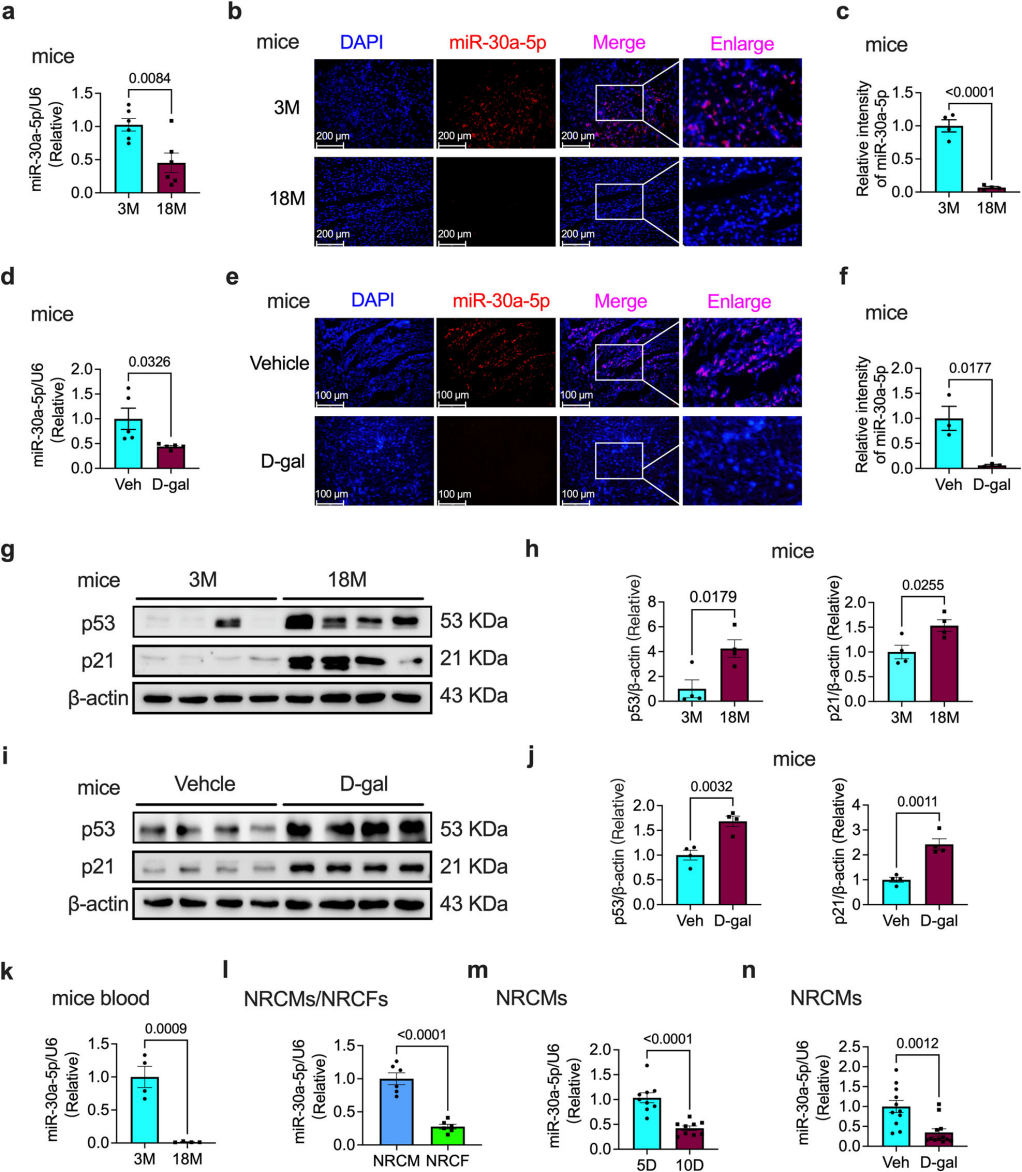
miR-30a-5p is downregulated in aging hearts and NRCMs. **a** qRT‒PCR analysis of miR-30a-5p expression in the heart tissues of 3- and 18-month-old WT mice (*n* = 6 in each group). Representative RNA-FISH images (**b**) and analysis (**c**) of miR-30a-5p levels in heart tissues of 3- and 18-month-old WT mice (*n* = 4 in each group). **d** qRT‒PCR analysis of miR-30a-5p levels in the hearts of vehicle- or D-gal-treated WT mice (*n* = 5 in each group). Representative RNA-FISH images (**e**) and analysis (**f**) of miR-30a-5p levels in the heart tissues of vehicle- or D-gal-induced mice (*n* = 3 in each group). Representative western blotting images (**g**) and analysis (**h**) of p53 and p21 expression in the heart tissues of 3- and 18-month-old WT mice (*n* = 4 in each group). Representative western blotting images (**i**) and analysis (**j**) of p53 and p21 protein levels in the heart tissues of the vehicle- or D-gal-treated mice (*n* = 4 in each group). **k** qRT‒PCR analysis of miR-30a-5p expression in the blood of 3- or 18-month-old mice (*n* = 4 in both groups). **l** qRT‒PCR analysis of miR-30a-5p expression in NRCMs and NRCFs (*n* = 6 in each group). **m** qRT‒PCR analysis of miR-30a-5p expression in 5- and 10-day-treated NRCMs (*n* = 9 in each group). **n** qRT‒PCR analysis of miR-30a-5p expression in vehicle- and D-gal-induced NRCMs (*n* = 10 in each group). The data are presented as the means ± standard errors. Statistical significance was assessed using t tests. WT wild-type, qRT‒PCR quantitative real-time polymerase chain reaction, RNA‒FISH RNA‒fluorescence in situ hybridization, veh vehicle, D‒gal D‒galactose, NRCMs neonatal rat cardiomyocytes, NRCFs neonatal rat cardiac fibroblasts.

To identify the role of miR-30a-5p in the aging heart, we examined miR-30a-5p expression in young (3-month-old) and old (18-month-old) mouse hearts. Using qPCR, we found that miR-30a-5p expression was significantly decreased in the hearts of aged mice (Fig. 1a). RNA fluorescence in situ hybridization (FISH) revealed similar results in young and aged myocardium (Fig. 1b, c). We also used a mouse model of D-gal-induced aging, a widely used inducer of senescence in animal or cell models^24–27^. The expression of miR-30a-5p was significantly reduced in the hearts of D-gal-treated mice, as detected by qPCR (Fig. 1d) and RNA-FISH (Fig. 1e, f). Furthermore, we investigated the expression of the senescence markers p53 and p21 in young and aging hearts. The expression of p53 and p21 was significantly increased in the hearts of 18-month-old (Fig. 1g, h) and D-gal-treated (Fig. 1i, j) mice. β-gal-positive cells were increased in the hearts of 18-month-old (Supplementary Fig. 1a, b) and D-gal-treated (Supplementary Fig. 1c, d) mice. Cardiac fibrosis (Supplementary Fig. 2a, b) and hypertrophy (Supplementary Fig. 2c, d) were also observed in both natural- and D-gal-induced aging hearts. In addition, we also tested the expression of miR-30a-5p in the blood of 3- and 18-month-old mice and found that its expression was significantly decreased in 18-month-old mice (Fig. 1k).

Cardiomyocytes and cardiac fibroblasts are the predominant cells in the heart. We isolated neonatal rat cardiomyocytes (NRCMs) and neonatal rat cardiac fibroblasts (NRCFs) and found that miR-30a-5p was expressed mainly in NRCMs but not in NRCFs (Fig. 1l). We then examined miR-30a-5p expression in naturally aged and D-gal-induced senescent NRCMs. Compared with that in 5-day-old NRCMs, the expression of miR-30a-5p exhibited a significant decrease in NRCMs after 10-day treatment compared to 5-day treatment (Fig. 1m), as well as in D-gal-treated NRCMs (Fig. 1n). In addition, we investigated the expression of miR-30a-5p in aged human cells. We found that miR-30a-5p expression was also downregulated in aging human umbilical vein endothelial cells (HUVECs) (Supplementary Fig. 3a) and D-gal-treated AC16 human cardiomyocytes (AC16) (Supplementary Fig. 3b) but D-gal had no effect on human cardiac fibroblasts (HCFs) (Supplementary Fig. 3c). These data suggest that miR-30a-5p may participate in cardiac senescence.

### miR-30a-5p knockout aggravates heart senescence

**Fig. 2:**
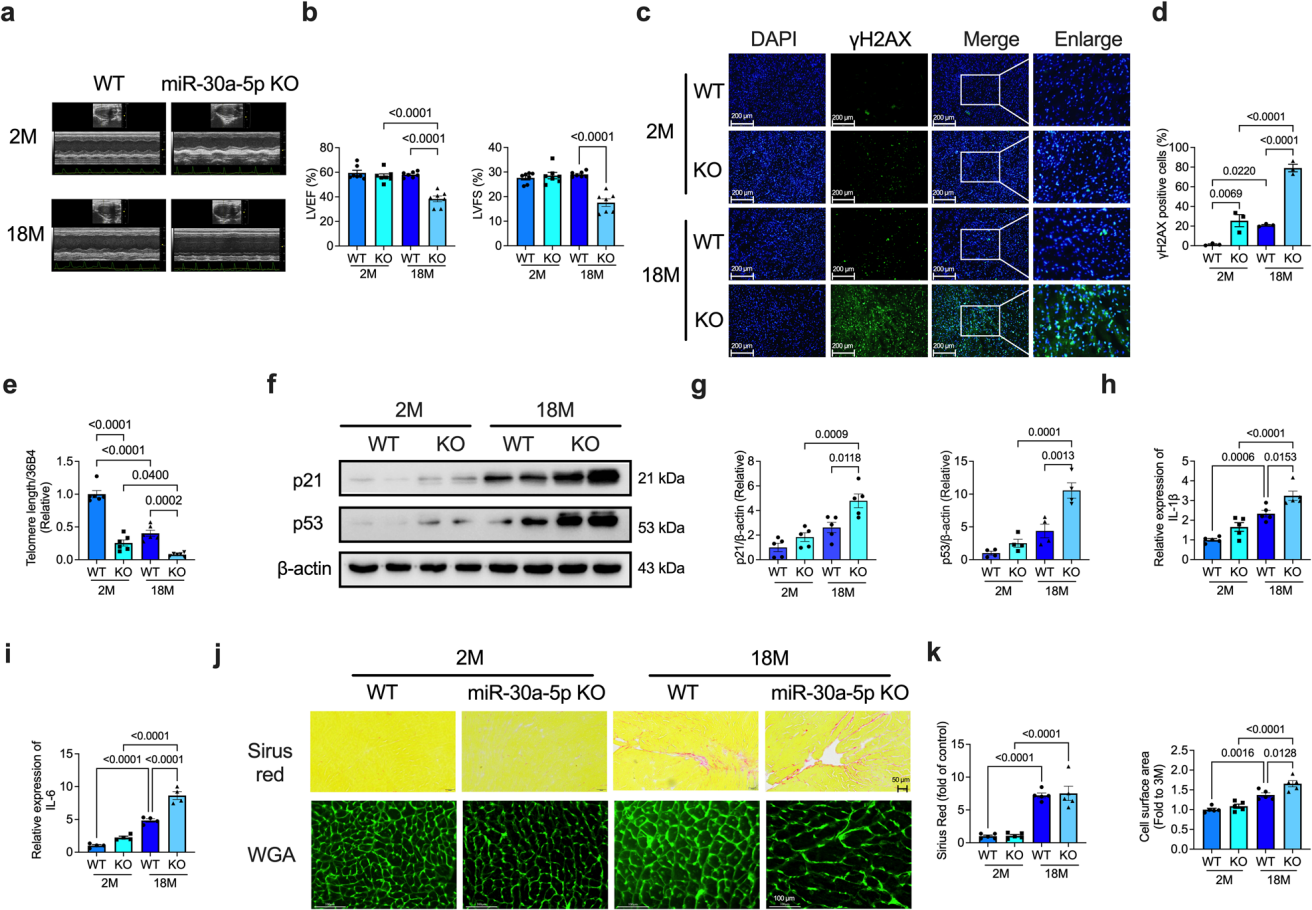
miR-30a-5p KO aggravates the aging-induced decrease in cardiac function and promotes senescence. Representative ECG images (**a**) and analysis (**b**) of LVEF and LVFS in 2- and 18-month-old WT and KO mice (*n* = 7 in each group). Representative images of immunofluorescence staining (**c**) and analysis (**d**) of γH2AX in heart tissues of 2- and 18-month-old WT and KO mice (scale bars=200 μm; *n* = 3 in each group). **e** qRT‒PCR analysis of telomere length in heart tissues of 2- and 18-month-old WT and KO mice (*n* = 6 in each group). Representative western blotting images (**f**) and analysis (**g**) of the expression of p21 (*n* = 5 in each group) and p53 (*n* = 4 in each group) in the heart tissues of 2- and 18-month-old WT and KO mice. qRT‒PCR analysis of myocardial IL-1β (**h**, *n* = 5 in each group) and IL-6 (**i**, *n* = 4 in each group) levels. Representative Picrosirius Red (scale bars = 50 μm) and wheat germ agglutinin (WGA, scale bars = 100 μm) staining images (**j**) and analysis (**k**) of the hearts of 2- and 18-month-old WT and KO mice (*n* = 5 in each group). The data are presented as the means ± standard errors. Statistical significance was assessed using one-way ANOVA. ECG echocardiography, WT wild-type, KO knockout, LVEF left ventricular ejection fraction, LVFS left ventricular fractional shortening, qRT‒PCR quantitative real-time polymerase chain reaction, γH2AX H2A histone family member X, WGA wheat germ agglutinin.

To examine the role of miR-30a-5p in heart aging, we constructed miR-30a-5p knockout (KO) mice. KO efficiency was determined via PCR and quantitative real-time polymerase chain reaction (qRT‒PCR), with significantly lower miR-30a-5p expression in the hearts of the KO mice than in those of their wild-type (WT) littermates (Supplementary Fig. 4a, b). We evaluated the role of miR-30a-5p KO in the hearts of naturally aged (18-month-old) KO and WT mice. Cardiac function in 2- and 18-month-old KO and WT mice was assessed using echocardiography. The left ventricular ejection fraction (LVEF) and left ventricular fractional shortening (LVFS) were significantly lower in aged KO mice than in their age-matched WT littermates (Fig. 2a, b). In addition, we tested the senescence marker H2A histone family member X (γH2AX) in myocardial tissues and found more γH2AX-positive cells in aged KO mice than in aged WT mice (Fig. 2c, d). Telomere length was also decreased in aged KO mice compared with young KO mice or aged WT mice (Fig. 2e). Moreover, p21 and p53 levels (Fig. 2f, g), β-gal-positive cells (Supplementary Fig. 5a, b), as well as myocardial IL-1β (Fig. 2h) and IL-6 (Fig. 2i) levels, were greater in aged KO mice than in aged WT mice. Cardiac hypertrophy was also promoted in aged KO mice but KO did not affect cardiac fibrosis (Fig. 2j, k).

The kidney and liver functions of the aging KO mice were examined. We found that KO did not affect kidney construction or the blood levels of uric acid (UA), creatinine (CR), or urea (UR) in both young and old mice (Supplementary Fig. 6a, b). The liver structure and functions, such as aspartate transaminase (AST) and alanine aminotransferase (ALT) levels and the AST/ALT ratio in the blood, were also not affected by KO (Supplementary Fig. 6c, d).

**Fig. 3:**
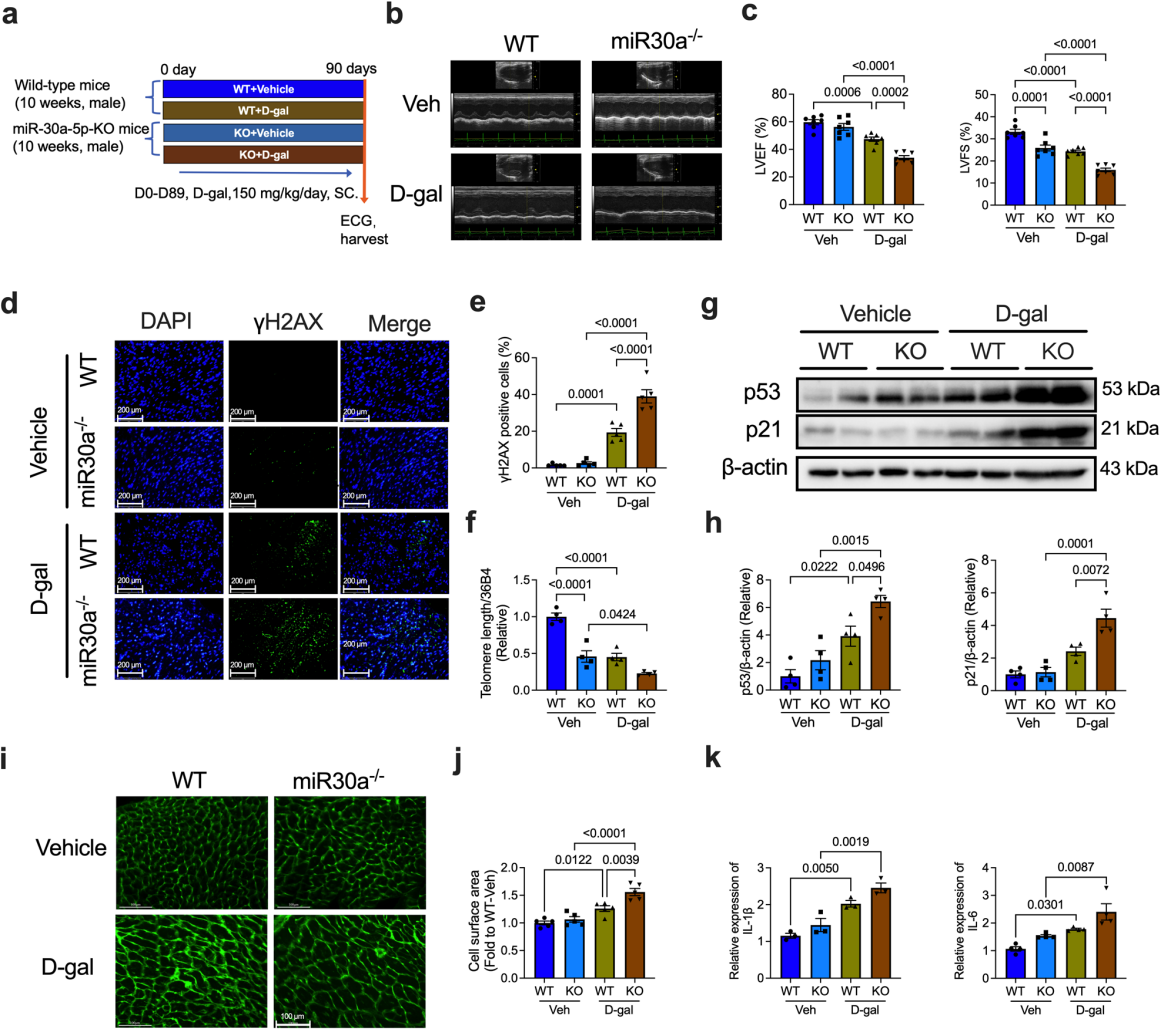
D-gal-induced heart senescence is aggravated in miR-30a-5p KO mice. **a** Schematic of the experimental timeline of D-gal-induced aging in WT and KO mice. Representative ECG images (**b**) and analysis (**c**) of the LVEF and LVFS of Veh- or D-gal-induced WT and KO mice (*n* = 7 in each group). Representative immunofluorescence staining (**d**) and analysis (**e**) of γH2AX in heart tissues of Veh- or D-gal-induced WT and KO mice (*n* = 4 in each group, scale bars = 200 μm). **f** qRT‒PCR analysis of telomere length in heart tissues from Veh- or D-gal-induced WT and KO mice (*n* = 6 in each group). Representative western blotting images (**g**) and analysis (**h**) of p53 and p21 expression in heart tissues of Veh- or D-gal-induced WT and KO mice (*n* = 4 in each group). Representative WGA staining images (**i**) and analysis (**j**) of the hearts of Veh- or D-gal-induced WT and KO mice (*n* = 5 in each group; scale bars = 100 μm). **k** qRT‒PCR analysis of myocardial IL-1β (*n* = 3 in each group) and IL-6 (*n* = 4 in each group) levels in Veh- or D-gal-induced WT and KO mice. The data are presented as the means ± standard errors. Statistical significance was assessed using one-way ANOVA. ECG echocardiography, WT wild-type, KO knockout, LVEF left ventricular ejection fraction, LVFS left ventricular fractional shortening, D-gal D-galactose, qRT‒PCR quantitative real‒time polymerase chain reaction, γH2AX H2A histone family member X, Veh vehicle, WGA wheat germ agglutinin.

Furthermore, we used D-gal-treated KO and WT littermate mice to validate the role of miR-30a-5p deficiency in D-gal-induced heart aging (Fig. 3a). Consistent with natural aging, D-gal-induced senescence also caused left ventricular dysfunction (Fig. 3b, c), increased the percentage of γH2AX-positive cells (Fig. 3d, e), shortened the telomere length (Fig. 3f), and upregulated p53 and p21 expression (Fig. 3g, h) in the cardiac tissues of the KO mice. KO also promoted D-gal-induced cardiac hypertrophy (Fig. 3i, j) and increased myocardial IL-1β and IL-6 levels (Fig. 3k), as well as the number of β-gal-positive cells (Supplementary Fig. 7a, b). In summary, miR-30a-5p deletion decreased cardiac function and telomere length and promoted senescence-related marker expression, indicating a significant role for miR-30a-5p in regulating heart aging.

### Cardiac-specific knockdown of miR-30a-5p decreases heart function and promotes heart senescence

**Fig. 4:**
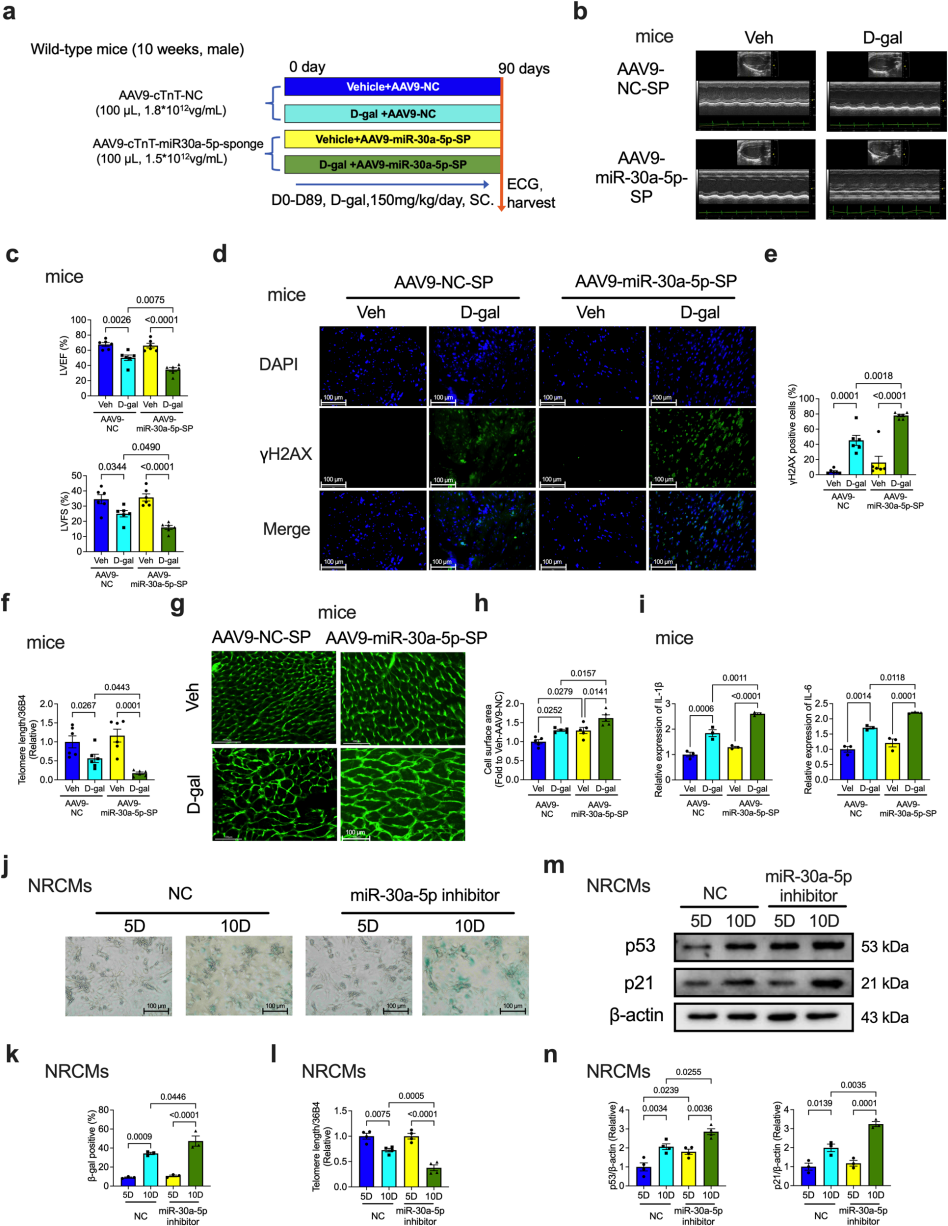
Cardiac-specific knockdown of miR-30a-5p aggravates D-gal-induced heart senescence. **a** Schematic of the experimental design of D-gal-induced aging in WT mice transfected with AAV9-NC-sponge or AAV9-miR-30a-5p-sponge. Representative ECG images (**b**) and analysis (**c**) of the LVEF and LVFS of Veh- or D-gal-induced mice transfected with AAV9-cTnT-NC-sponge or AAV9-cTnT-miR-30a-5p-sponge (*n* = 6 in each group). Representative immunofluorescence staining (**d**) and analysis (**e**) of γH2AX in heart tissues of Veh- or D-gal-induced aging in mice treated with AAV9-cTnT-NC-sponge or AAV9-cTnT-miR-30a-5p-sponge (*n* = 6 in each group, scale bars = 100 μm). **f** qRT‒PCR analysis of telomere length in heart tissues of Veh- or D-gal-induced aging in mice treated with AAV9-cTnT-NC-sponge or AAV9-cTnT-miR-30a-5p-sponge (*n* = 6 in each group). Representative WGA staining images (**g**) and analysis (**h**) of the hearts of Veh- or D-gal-induced aging in mice treated with AAV9-cTnT-NC-sponge or AAV9-cTnT-miR-30a-5p-sponge (*n* = 5 in each group, scale bars = 100 μm). **i** qRT‒PCR analysis of myocardial IL-1β and IL-6 levels in Veh- or D-gal-induced aging in mice treated with AAV9-cTnT-NC-sponge or AAV9-cTnT-miR-30a-5p-sponge (*n* = 3 in each group). Representative SA-β-gal staining images (**j**) and analysis (**k**) of NRCMs transfected with control inhibitor or miR-30a-5p inhibitor cultured for 5 or 10 days (*n* = 3 in each group, scale bars = 100 μm). **l** qRT‒PCR analysis of telomere length in NRCMs cultured for 5 or 10 days and transfected with control inhibitor or miR-30a-5p inhibitor (*n* = 6 in each group). Representative western blotting images (**m**) and analysis (**n**) of p53 (*n* = 4 in each group) and p21 (*n* = 3 in each group) expression in NRCMs transfected with the control inhibitor or the miR-30a-5p inhibitor and cultured for 5 or 10 days. The data are presented as the means ± standard errors. Statistical significance was assessed using one-way ANOVA. WT wild-type, LVEF left ventricular ejection fraction, LVFS left ventricular fractional shortening, Veh vehicle, D-gal D-galactose, ECG echocardiography, qRT‒PCR quantitative real-time polymerase chain reaction, γH2AX H2A histone family member X, NRCMs neonatal rat cardiomyocytes.

Given that miR-30a-5p was systemically knocked out in these KO mice, we further applied cardiac-specific knockdown of miR-30a-5p to validate its role in the aging heart. We injected adeno-associated virus (AAV)9-miR-30a-5p-sponge and AAV9-control on day 0 and induced senescence by administering D-gal daily from day 0 to day 89. Three weeks after the AAV9 injection, the efficiency of AAV9 infection in the heart was evaluated on the basis of luciferase activity (the AAV9 construct contained a luciferase segment) via a Caliper IVIS Lumina II in vivo imaging system (Supplementary Fig. 8a). The knockdown efficiency of miR-30a-5p was confirmed via qRT‒PCR, which revealed significantly lower miR-30a-5p expression (Supplementary Fig. 8b) in AAV9-miR-30a-5p-transfected hearts than in AAV9-negative control (NC)-transfected hearts. After D-gal treatment, echocardiography was performed to assess cardiac function and senescence-related factors (Fig. 4a). In AAV9-NC (AAV9-NC-SP)-transfected D-gal-treated mice, left ventricular function decreased slightly (Fig. 4b, c). Compared with AAV9-NC treatment combined with D-gal treatment, miR-30a-5p-sponge treatment (AAV9-miR-30a-5p-SP) significantly reduced cardiac function (Fig. 4b, c). The percentage of γH2AX-positive cells in heart tissue was also markedly greater after AAV9-miR-30a-5p sponge infection than after AAV9-NC infection under D-gal induction (Fig. 4d, e). The D-gal-induced decrease in telomere length was further decreased by the miR-30a-5p sponge (Fig. 4f). A cardiac-specific miR-30a-5p sponge also promoted D-gal-induced cardiac hypertrophy (Fig. 4g, h) and myocardial IL-1β and IL-6 levels (Fig. 4i), along with an increase in β-gal-positive cells (Supplementary Fig. 9a, b).

Additionally, we validated the role of miR-30a-5p inhibition in naturally aged NRCMs, and similar effects were observed in these cells. Compared with 5 days of treatment, inhibition of miR-30a-5p increased the ratio of senescence-associated β-galactosidase (SA-β-gal)-positive cardiomyocytes (Fig. 4j, k), decreased telomere length (Fig. 4l), and increased p53 and p21 expression (Fig. 4m, n) at 10 days.

### Cardiac-specific overexpression of miR-30a-5p alleviates D-gal-induced cardiac senescence

Considering that miR-30a-5p KO aggravated cardiac dysfunction and senescence, we hypothesized that miR-30a-5p overexpression may have a protective effect on heart aging. Next, we evaluated whether cardiac-specific overexpression of miR-30a-5p may have a therapeutic effect on D-gal-induced cardiac dysfunction and senescence. The efficiency of AAV9 infection in the heart was confirmed using a Caliper IVIS Lumina II in vivo imaging system (Supplementary Fig. 10a), and the overexpression efficiency of miR-30a-5p was validated by qRT-PCR, which revealed significantly increased expression in the overexpression group (Supplementary Fig. 10b).

**Fig. 5:**
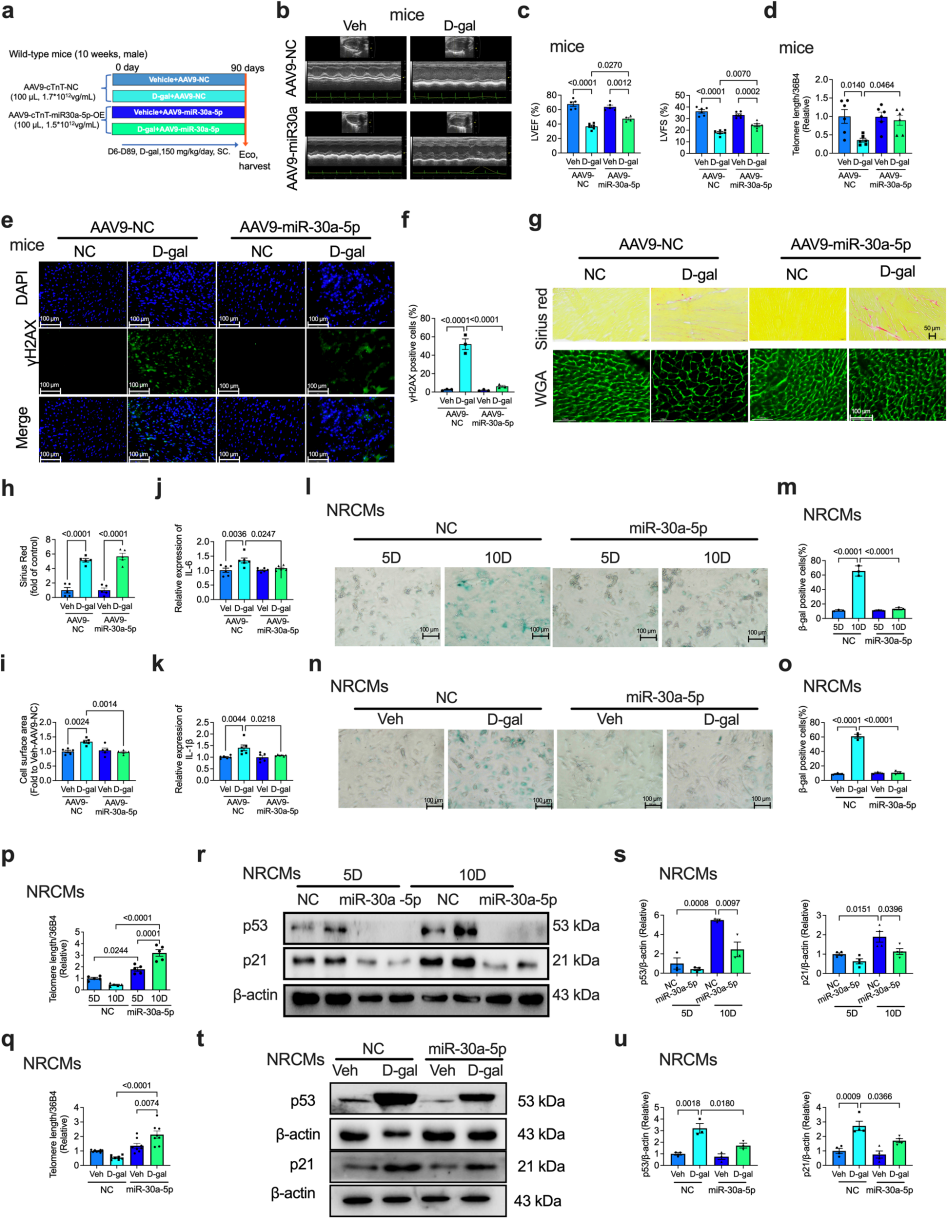
Cardiac-specific overexpression of miR-30a-5p in WT mice alleviates D-gal-induced cardiac senescence. **a** Schematic of the experimental timeline of D-gal-induced aging in mice transfected with AAV9-cTnT-control (NC) or AAV9-cTnT-miR-30a-5p-overexpressing (OE) vectors. Representative ECG images (**b**) and analysis (**c**) of LVEF and LVFS in NC or OE WT mice induced with Veh or D-gal (*n* = 6 in each group). **d** qRT‒PCR analysis of telomere length in the heart tissues of NC or OE WT mice induced with Veh or D-gal (*n* = 6 in each group). Representative immunofluorescence staining (**e**) and analysis (**f**) of γH2AX in the heart tissues of NC or OE WT mice induced with Veh or D-gal (*n* = 3 in each group; scale bars = 100 μm). Representative Picrosirius Red and WGA staining images (**g**) and analysis (**h** and **i**) of the hearts of NC or OE WT mice induced with Veh or D-gal (*n* = 5 in each group; scale bars = 50 μm or 100 μm). qRT‒PCR analysis of myocardial IL-1β (**j**) and IL-6 (**k**) levels in NC or OE mice induced with Veh or D-gal (*n* = 6 in each group). Representative SA-β-gal staining images (**l**) and analysis (**m**) of NRCMs cultured for 5 or 10 days and transfected with control or miR-30a-5p mimics (*n* = 3 per group; scale bars = 100 μm). Representative SA-β-gal staining images (**n**) and analysis (**o**) of NRCMs transfected with control or miR-30a-5p mimics in the presence or absence of D-gal (*n* = 3 per group, scale bars = 100 μm). **p** qRT‒PCR analysis of telomere length in NRCMs cultured for 5 or 10 days and transfected with control mimics or miR-30a-5p mimics (*n* = 5 in each group). **q** qRT‒PCR analysis of telomere length in NRCMs transfected with control or miR-30a-5p mimics in the presence or absence of D-gal (*n* = 8 in each group). Representative western blotting images (**r**) and analysis (**s**) of p53 (*n* = 3 in each group) and p21 (*n* = 4 in each group) expression in NRCMs cultured for 5 or 10 days and transfected with control or miR-30a-5p mimics. Representative western blotting images (**t**) and analysis (**u**) of p53 (*n* = 3 in each group) and p21 (*n* = 4 in each group) expression in NRCMs transfected with control or miR-30a-5p mimics in the presence or absence of D-gal. The data are presented as the means ± standard errors. Statistical significance was assessed using one-way ANOVA. NC control, OE overexpression, ECG echocardiography, LVEF left ventricular ejection fraction, LVFS left ventricular fractional shortening, D-gal D-galactose, WT wild-type, qRT‒PCR quantitative real-time polymerase chain reaction, γH2AX H2A histone family member X, β-gal β-galactosidase, Veh vehicle, NRCMs neonatal rat cardiomyocytes, SA-β-gal senescence-associated β-galactosidase.

The experimental design of the D-gal-treated aging mouse model is shown in Fig. 5a. Cardiac function based on the parameters LVEF and LVFS, was impaired in D-gal-treated AAV9-NC-transfected mice but was rescued by miR-30a-5p overexpression in the heart (Fig. 5b, c). The D-gal-mediated decrease in telomere length was reversed by AAV9-miR-30a-5p (Fig. 5d). Moreover, the D-gal-induced increase in the number of γH2AX-positive cells was suppressed by miR-30a-5p overexpression (Fig. 5e, f). Cardiac fibrosis was not suppressed by AAV9-miR-30a-5p (Fig. 5g, h). However, D-gal-induced cardiac hypertrophy (Fig. 5g, i), myocardial IL-1β and IL-6 levels (Fig. 5j, k), and β-gal-positive cells (Supplementary Fig. 11a, 11b) were significantly reduced by the overexpression of miR-30a-5p.

The role of miR-30a-5p overexpression in vitro was also investigated in naturally aged and D-gal-treated NRCMs. The overexpression of miR-30a-5p also suppressed the 10-day- (Fig. 5l, m) and D-gal- (Fig. 5n, o)-induced increase in the number of SA-β-gal-positive cells. In both 10-day (Fig. 5p) and D-gal-treated (Fig. 5q) cells, the telomere length was reduced in NC-transfected NRCMs but was significantly increased by miR-30a-5p overexpression. The expression of the senescence-related proteins p53 and p21 was also assessed in NRCMs subjected to natural aging- and D-gal-induced senescence. The 10-day-induced (Fig. 5r, s) and D-gal-induced (Fig. 5t, u) upregulation of p53 and p21 was significantly decreased by the miR-30a-5p mimics. These data suggest that miR-30a-5p overexpression effectively rescued cardiac senescence both in vitro and in vivo.

We further investigated the role of the overexpression of miR-30a-5p in D-gal-induced senescent neonatal rat cardiac fibroblasts (NRCFs). The D-gal-induced decrease in telomere length was not reversed by miR-30a-5p in NRCFs (Supplementary Fig. 12a). D-gal-induced β-gal-positive cells were not significantly improved by miR-30a-5p (Supplementary Fig. 12b, c). The expression of p53 and p21 was also unaffected by miR-30a-5p (Supplementary Fig. 12d, e).

### TP53INP1 is involved in miR-30a-5p-regulated heart aging

**Fig. 6:**
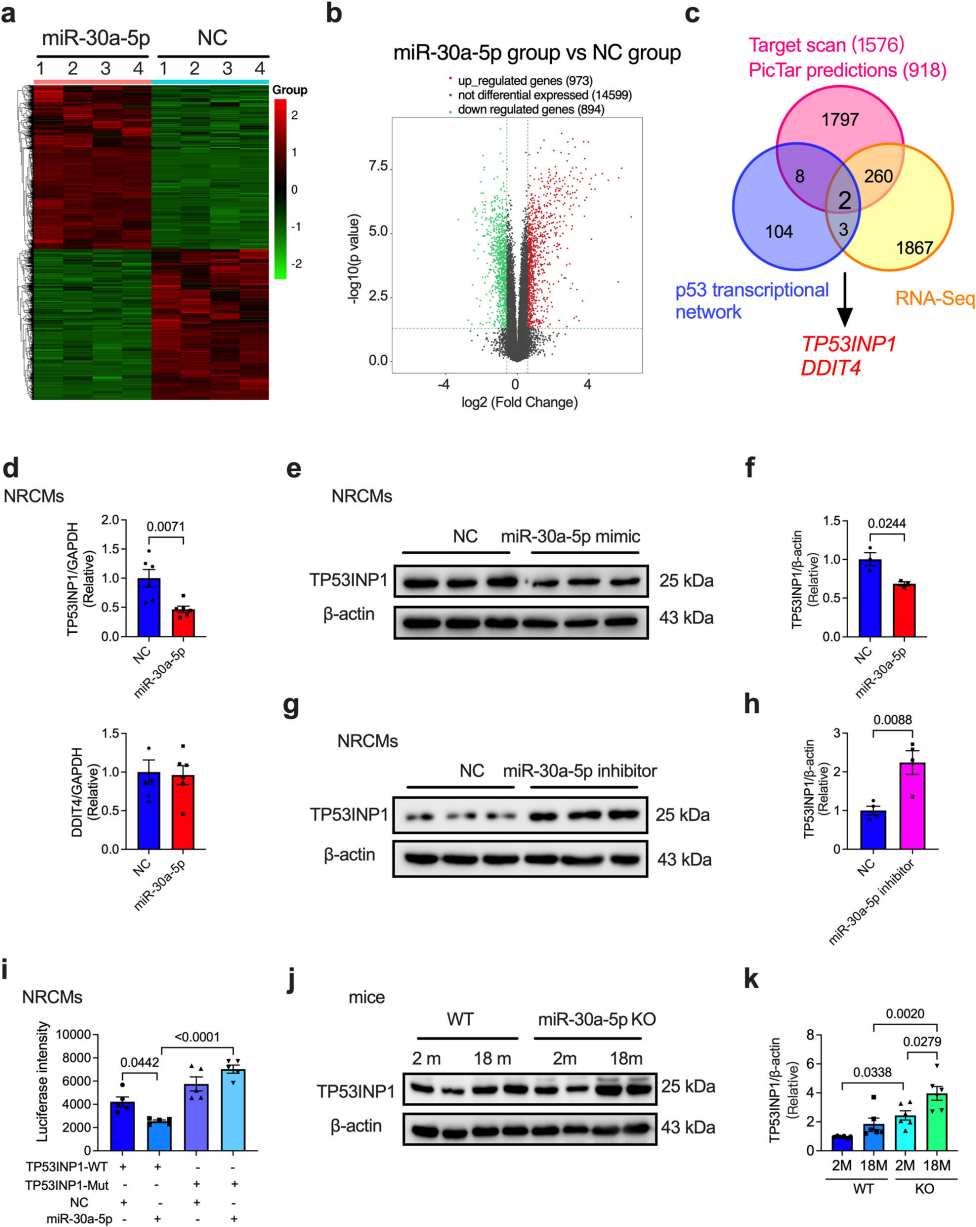
TP53INP1 is regulated by miR-30a-5p. Heatmap (**a**) and volcano plot (**b**) of RNA sequence data from NRCMs transfected with control mimics or miR-30a-5p mimics. **c** Combinations of RNA sequencing (1867), target prediction (TargetScan and PicTar predictions) (1797), and p53-transcriptional gene network (104) data identified *Trp53inp* and *Ddit4* as potential targets of miR-30a-5p. **d** qRT‒PCR analysis of *Tp53inp1* and *Ddit4* in NRCMs transfected with NC or miR-30a-5p mimics (*n* = 6 in each group). Representative western blotting images (**e**) and quantitative analysis (**f**) of TP53INP1 expression in NRCMs transfected with NC or miR-30a-5p mimics (*n* = 3 in each group). Representative western blotting (**g**) and quantitative analysis (**h**) of TP53INP1 expression in NRCMs transfected with NC or the miR-30a-5p inhibitor (*n* = 4 in each group). **i** Luciferase activity assay of NRCMs cotransfected with NC or miR-30a-5p mimics and WT or mutant TP53INP1 (*n* = 5 in each group). Representative western blots (**j**) and quantitative analysis (**k**) of TP53INP1 expression in the hearts of 2- or 18-month-old WT or KO mice (*n* = 6 in each group). The data are presented as the means ± standard errors. Statistical significance was assessed using one-way ANOVA or t tests. NRCMs neonatal rat cardiomyocytes, qRT‒PCR quantitative real-time polymerase chain reaction, WT wild-type, KO knockout, NC negative control.

To further investigate the mechanisms underlying the regulation of heart senescence by miR-30a-5p, we examined the mRNA expression of NRCMs transfected with control or miR-30a-5p mimics. The results revealed that 973 genes were upregulated and 894 were downregulated, as shown in the heatmap (Fig. 6a) and volcano plot (Fig. 6b). We combined the RNA-seq results (1867 altered genes), the predicted target genes of miR-30a-5p via TargetScan (1576 predicted target genes) and PicTar (918 predicted target genes), and the p53-transcriptional network (104 genes). We identified that *Tp53inp1* (encoding the tumor protein p53 inducible nuclear protein 1 [TP53INP1]) and *Ddit4* may be potential targets of miR-30a-5p-regulated senescence (Fig. 6c). The mRNA levels of *Tp53inp1* and *Ddit4* were validated by qRT‒PCR in control- and miR-30a-5p mimic-transfected NRCMs. The miR-30a-5p mimic-transfected cells presented a significant decrease in *Tp53inp1* but not *Ddit4* (Fig. 6d).

Next, we tested whether the TP53INP1 protein level was influenced by miR-30a-5p. TP53INP1 levels were significantly decreased by the miR-30a-5p mimics (Fig. 6e, f) and increased by the inhibitor (Fig. 6g, h). However, DDIT4 expression was not affected by the miR-30a-5p mimics (Supplementary Fig. 13a). Furthermore, we constructed two dual-luciferase reporter genes that expressed WT or mutant TP53INP1 and cotransfected them with control mimics or miR-30a-5p mimics to determine whether TP53INP1 is a direct target of miR-30a-5p. The luciferase activity of WT TP53INP1, but not that of mutant TP53INP1, was suppressed by transfection with miR-30a-5p (Fig. 6i), indicating that miR-30a-5p directly regulates TP53INP1. We also examined the luciferase activity of DDIT4, which revealed that it was not significantly affected by the transfection of miR-30a-5p (Supplementary Fig. 13b). Additionally, TP53INP1 levels were examined in 2- and 18-month-old WT and KO mice. Higher expression of TP53INP1 was observed in 18-month-old WT mice than in 2-month-old WT mice, and TP53INP1 expression was significantly further upregulated in 18-month-old KO mice (Fig. 6j, k). These data suggest that TP53INP1 is a direct target of miR-30a-5p in the regulation of cardiac aging.

We explored the role of TP53INP1 in senescent NRCMs. Small interfering RNA (siRNA) targeting *Tp53inp1* was transfected into NRCMs. A significant decrease in TP53INP1 expression was observed in *Tp53inp1* siRNA-transfected cells compared with that in scrambled siRNA-transfected cells (Supplementary Fig. 14a, b). The levels of p53 were also reduced by *Tp53inp1* knockdown (Supplementary Fig. 14a, b), suggesting that p53 is regulated by TP53INP1. The p53 and p21 levels were also decreased in *Tp53inp1* siRNA-transfected naturally aged NRCMs (10-day treatment; Supplementary Fig. 14c, d). Furthermore, telomere length in 10-day-treated NRCMs was increased in *Tp53inp1* siRNA-transfected NRCMs (Supplementary Fig. 14e), and the number of SA-β-gal-positive cells induced by natural aging (10-day treatment) was reduced in *Tp53inp1* siRNA-transfected NRCMs (Supplementary Fig. 14f, g). These data suggest that TP53INP1 is an important regulator of cardiomyocyte senescence.

### miR-30a-5p deficiency promotes TP53INP1 transition and SUMOylation

**Fig. 7:**
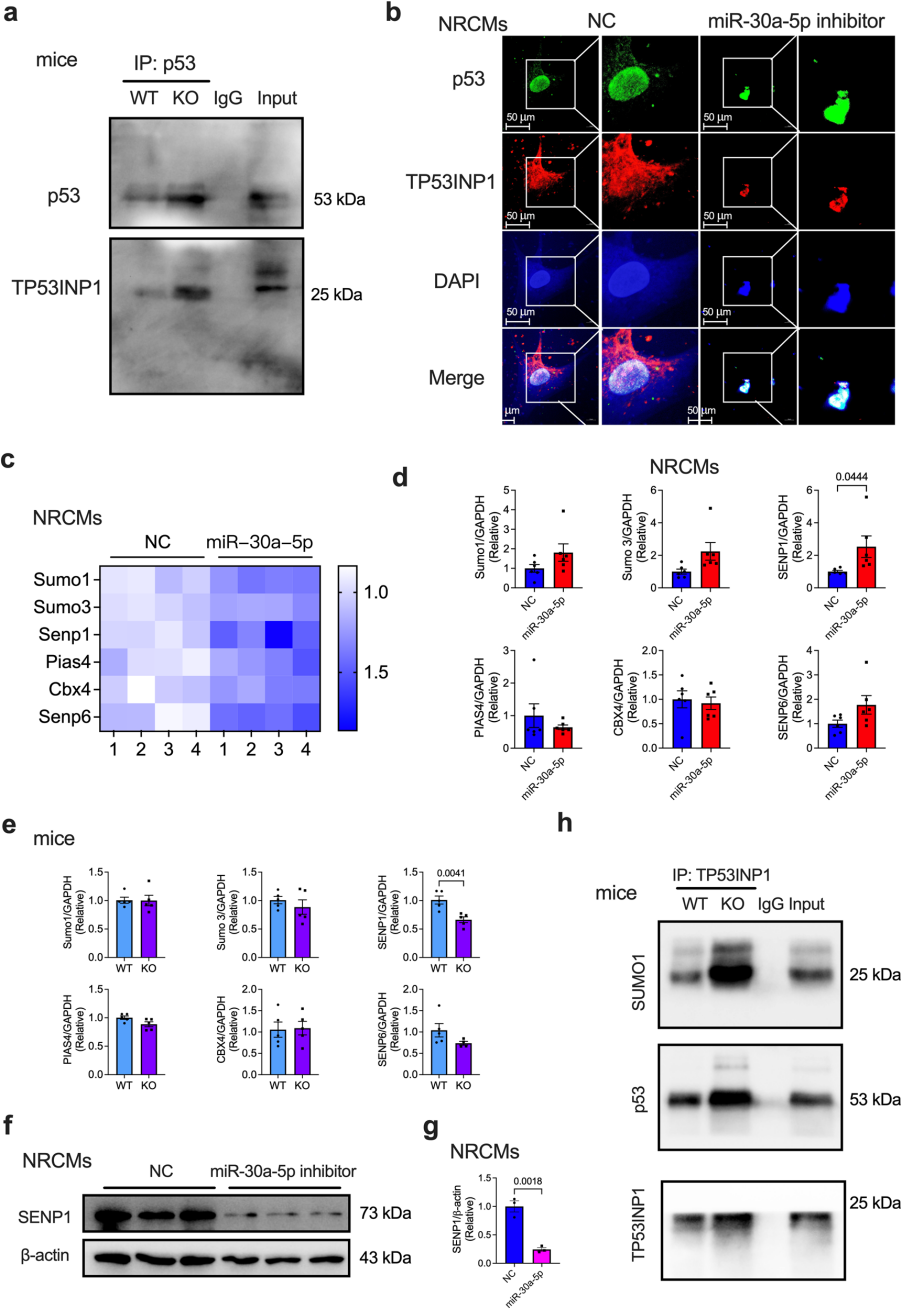
Deficiency of miR-30a-5p promotes the interaction of TP53INP1 with p53 and TP53INP1 SUMOylation. **a** Representative immunoprecipitation detection of the interaction between p53 and TP53INP1 in the heart tissues of WT and KO mice. **b** Representative immunofluorescence images of p53 (green) and TP53INP1 (red) in NRCMs transfected with the control or miR-30a-5p inhibitor (scale bars = 50 μm). DAPI (blue) was used to counterstain the nucleus. **c** Heatmap of SUMOylation-related genes from RNA-seq data of NRCMs transfected with control or miR-30a-5p mimics. **d** qRT‒PCR analysis of SUMOylation-related genes in NRCMs transfected with control or miR-30a-5p mimics (*n* = 6 in each group). **e** qRT‒PCR analysis of SUMOylation-related genes in heart samples from WT and KO mice (*n* = 5 in each group). Representative western blotting images (**f**) and quantitative analysis (**g**) of SENP1 in NRCMs transfected with control or miR-30a-5p inhibitor (*n* = 3 in each group). **h** Representative immunoprecipitation blot showing the interaction of TP53INP1 with SUMO1 and p53 in the hearts of WT and KO mice. The data are presented as the means ± standard errors. Statistical significance was assessed using one-way ANOVA. WT wild-type, KO knockout, NC negative control, NRCMs neonatal rat cardiomyocytes, qRT‒PCR quantitative real‒time polymerase chain reaction.

Because TP53INP1 is a positive modulator of p53 transcriptional activity^28^, we further explored the underlying mechanism of the miR-30a-5p-regulated relationship between TP53INP1 and p53. We first tested the interaction between TP53INP1 and p53 and found a significant increase in their interaction in the hearts of miR-30a-5p-KO mice (Fig. 7a). The interaction between p53 and TP53INP1 was validated in NRCMs transfected with the miR-30a-5p inhibitor. As shown in Fig. 7b, TP53INP1 was mainly located in the cytoplasm and cell nucleus, whereas p53 was localized in the nucleus. After NRCMs were transfected with the miR-30a-5p inhibitor, most TP53INP1 translocated from the cytoplasm to the nucleus (Fig. 7b and Supplementary Fig. 15). Our previous RNA-seq results revealed that six genes related to SUMOylation (*Sumo1*, *Sumo3*, *Senp1*, *Pias4*, *Cbx4*, and *Senp6*) were significantly upregulated in miR-30a-5p-overexpressing NRCMs (Fig. 7c). Thus, we determined their mRNA levels in NRCMs transfected with the miR-30a-5p mimics, as well as in WT and KO mice. The deSUMOylation enzyme SUMO-specific peptidase 1 (*Senp1*) was significantly upregulated by the miR-30a-5p mimics (Fig. 7d) and downregulated in the KO hearts (Fig. 7e), which was validated by the SENP1 protein levels in the miR-30a-5p inhibitor-transfected NRCMs (Fig. 7f, g). These data indicate that miR-30a-5p deficiency may promote SUMOylation by regulating the deSUMOylation enzyme SENP1. Thus, we determined the SUMOylation of TP53INP1 in the hearts of KO and WT mice. SUMO1 SUMOylation of TP53INP1 in KO mice was significantly greater than that in WT mice (Fig. 7h), indicating that the miR-30a-5p deficiency-induced increase in TP53INP1 expression was also mediated by increased SUMOylation of TP53INP1. In summary, these results indicate that miR-30a-5p deficiency promotes TP53INP1 translocation from the cytoplasm to the nucleus, facilitating SUMO1 SUMOylation of TP53INP1.

Furthermore, we constructed wild-type TP53INP1 via site-directed mutagenesis to change the putative SUMOylated lysine 113 residue to the arginine residue of TP53INP1 (TP53INP1^K113R^ mutant), constructed a SUMO1 plasmid, and transfected the resulting plasmid into AC16 cardiomyocytes. The findings revealed that when cells were transfected with WT TP53INP1, SUMO1 expression increased (Supplementary Fig. 16a). However, when cotransfected with the WT and K113R SUMOylation-deficient mutant of TP53INP1, the SUMO1 level decreased (Supplementary Fig. 16a), indicating that the K113 residue is the key site for TP53INP1 SUMOylation. We further investigated the effect of the TP53INP1^K113R^ mutant on miR-30a-5p-regualted cardiomyocyte aging. The findings showed that the miR-30a-5p-promoted increased in telomere length was notably decreased after the cells were transfected with the TP53INP1^K113R^ mutant (Supplementary Fig. 16b). Moreover, the decreased expression of p53 and p21 caused by miR-30a-5p was also dynamically restored by transfection with the TP53INP1^K113R^ mutant (Supplementary Fig. 16c, d). These results indicate that the TP53INP1 SUMOylation site is critical for miR-30a-5p-modulated cardiomyocyte aging.

In addition, we determined the mechanism by which miR-30a-5p regulates telomere length. We found that the expression of TERT and TERF2, two crucial telomere length-regulating enzymes, was regulated by miR-30a-5p, with decreased levels in miR-30a-5p inhibitor-transfected NRCMs (Supplementary Fig. 17a and s17b) and increased levels in miR-30a-5p mimic-transfected NRCMs (Supplementary Figs. 17c, d). Moreover, the decreased expression of *Tert* and *Terf2* in naturally aged (10-day-old) NRCMs was partially prevented by the miR-30a-5p mimics (Supplementary Fig. 17e, f), suggesting that miR-30a-5p affects telomere length by regulating TERT and TERF2 expression.

### Knockdown of TP53INP1 rescues miR-30a-5p KO-aggravated heart senescence

**Fig. 8:**
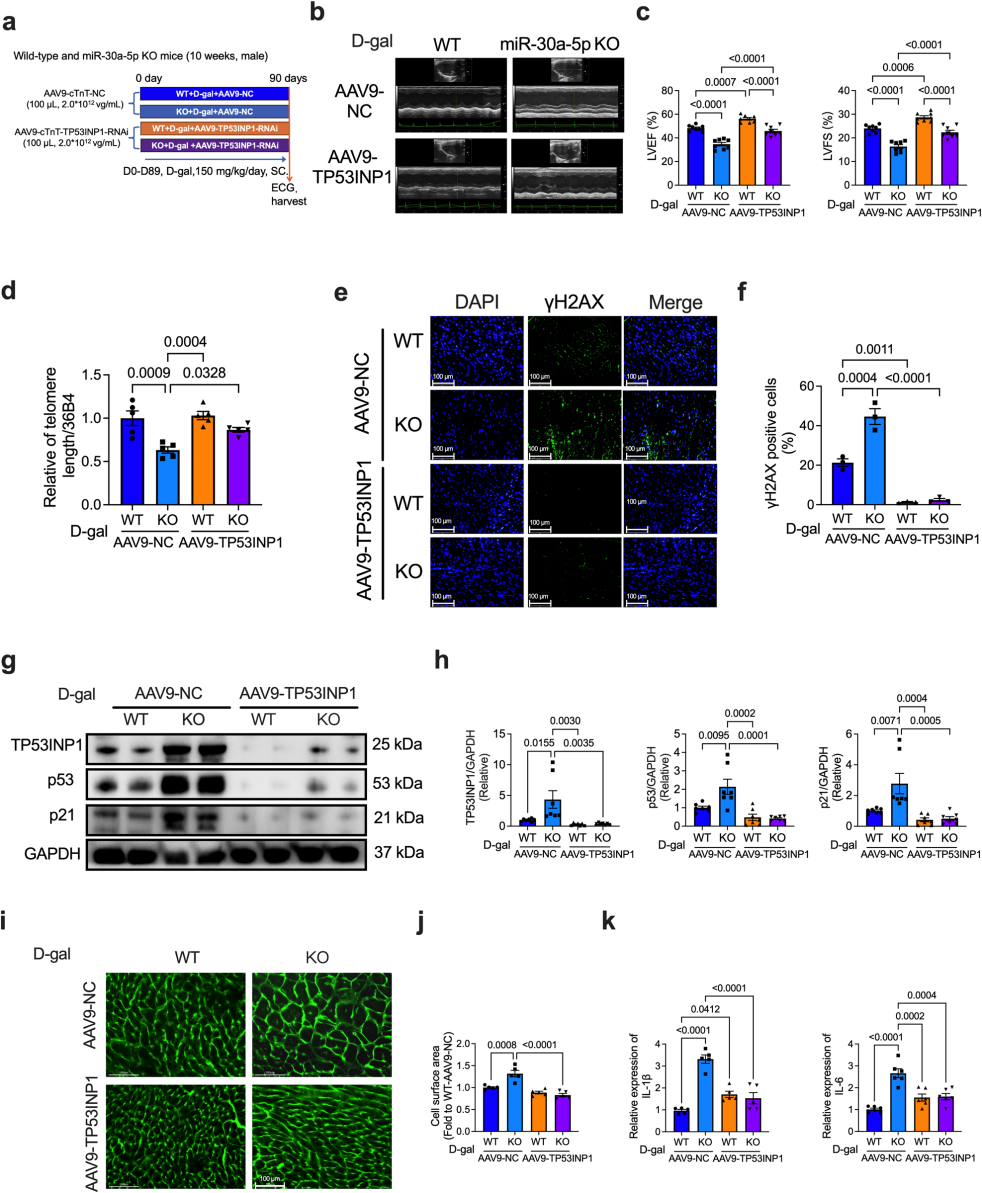
TP53INP1 deficiency attenuates miR-30a-5p KO-aggravated heart senescence. **a** Schematic of the experimental timeline of the D-gal-induced aging model in WT and KO mice transfected with AAV9-cTnT-control- or AAV9-cTnT-TP53INP1-RNAi. Representative ECG images (**b**) and analysis (**c**) of the LVEF and LVFS of D-gal-induced aged WT or KO mice transfected with AAV9-cTnT-control- or AAV9-cTnT-TP53INP1-RNAi (*n* = 8 in each group). **d** qRT‒PCR analysis of telomere length in heart tissues of AAV9-control- or AAV9-TP53INP1-RNAi-treated WT or KO mice induced with D-gal (*n* = 5 in each group). Representative immunofluorescence staining (**e**) and analysis (**f**) of γH2AX in heart tissues of AAV9-cTnT-control- or AAV9-cTnT-TP53INP1-RNAi-treated WT or KO mice induced with D-gal (*n* = 3 in each group; scale bars = 100 μm). Representative western blotting images (**g**) and analysis (**h**) of TP53INP1 (*n* = 7 in each group), p53 (*n* = 7 in each group), and p21 (*n* = 7 in each group) expression in heart tissues of AAV9-cTnT-control- or AAV9-cTnT-TP53INP1-RNAi-transfected WT or KO mice induced with D-gal. Representative WGA staining images (**i**) and analysis (**j**) of the hearts of AAV9-cTnT-control- or AAV9-cTnT-TP53INP1-RNAi-transfected WT or KO mice induced with D-gal (*n* = 5 in each group, scale bars = 100 μm). **k** qRT‒PCR analysis of myocardial IL-1β (*n* = 5 in each group) and IL-6 (*n* = 6 in each group) levels in AAV9-cTnT-control- or AAV9-cTnT-TP53INP1-RNAi-transfected WT or KO mice induced with D-gal. The data are presented as the means ± standard errors. Statistical significance was assessed using one-way ANOVA. WT wild-type, KO knockout, ECG echocardiography, LVEF left ventricular ejection fraction, LVFS left ventricular fractional shortening, D-gal D-galactose, qRT‒PCR quantitative real-time polymerase chain reaction, γH2AX H2A histone family member X.

To validate the in vivo effect of TP53INP1 on miR-30a-5p KO-aggravated heart senescence, we constructed an AAV9 carrying *Tp53inp1*-RNAi that can induce cardiac-specific knockdown of TP53INP1. AAV9-control (AAV9-NC)- or AAV9-TP53INP1-RNAi (AAV9-TP53INP1)-injected KO mice and their WT littermates were subjected to D-gal treatment for 3 months to induce senescence in vivo (Fig. 8a). We subsequently examined LV function via echocardiography and found that the D-gal-induced decreases in LVEF and LVFS in KO mice were significantly reversed by treatment with AAV9-TP53INP1 but not with AAV9-NC (Fig. 8b, c). In addition, the D-gal-induced decrease in telomere length in the hearts of the KO mice was prevented by AAV9-TP53INP1 (Fig. 8d), and the increase in the number of γH2AX-positive cells in the hearts of the KO mice was suppressed by TP53INP1 knockdown in the presence of D-gal (Fig. 8e, f). Furthermore, after D-gal treatment, the miR-30a-5p KO-induced increases in TP53INP1, p53, and p21 expression were inhibited by AAV9-TP53INP1-RNAi (Fig. 8g, h). These results demonstrated that the D-gal-induced decrease in cardiac function and senescence in miR-30a-5p-deficient mice can be reversed to some extent by the knockdown of TP53INP1. Furthermore, TP53INP1 silencing considerably alleviated D-gal-induced cardiac hypertrophy (Fig. 8i, j), myocardial IL-1β and IL-6 levels (Fig. 8k), and β-gal-positive cells (Supplementary Fig. 18a, b) in the KO mice.

## Discussion

**Fig. 9:**
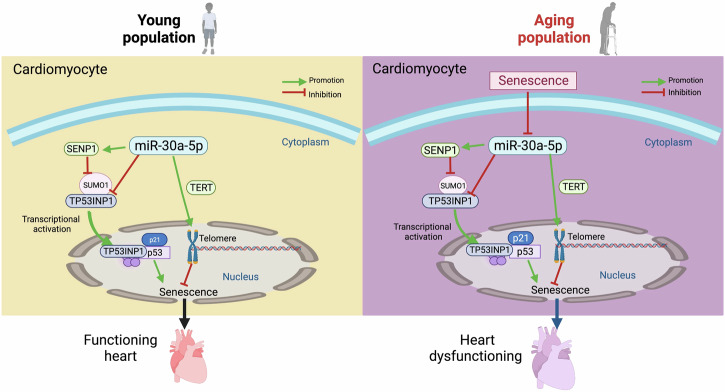
Graphic image. miR-30a-5p was downregulated in the hearts and cardiomyocytes of natural and D-galactose-induced aged mice. miR-30a-5p deficiency aggravated cardiac senescence, whereas cardiac-specific overexpression of miR-30a-5p protected against aging-induced cardiac dysfunction and senescence. TP53INP1 has been identified as a target of miR-30a-5p in the induction of cardiac senescence through its interaction with nuclear p53 via SUMOylation.

Aging is a well-established contributing factor to HF and the leading cause of death worldwide. In the present study, we demonstrated a novel mechanism mediating heart aging via miR-30a-5p-regulated TP53INP1 function. As shown in Fig. 9, the key findings of the study are as follows: 1) miR-30a-5p expression is decreased in the naturally senescent and D-gal-treated hearts of WT mice and NRCMs. 2) Mice lacking miR-30a-5p are prone to aggravated naturally aging- or D-gal-induced heart senescence, accompanied by cardiac dysfunction and telomere length shortening, as well as increases in γH2AX, p21, and p53 expression. 3) Cardiac-specific miR-30a-5p overexpression in WT mice alleviates D-gal-induced heart aging, reduces the number of γH2AX-positive cells, and increases p53 and p21 expression. 4) Mechanistically, TP53INP1 is a target of miR-30a-5p by regulating its translocation to the nucleus and its SUMOylation. 5) Cardiac-specific knockdown of TP53INP1 partially prevented miR-30a-5p KO-aggravated heart dysfunction and senescence.

Several studies have investigated the impact of miR-30a on cardiomyocyte and heart function. miR-30a-5p has been identified as a miRNA associated with blood pressure^29^, acute MI, and HF^19,20^. Moreover, miR-30a regulates Ang II-induced cardiomyocyte autophagy^30^, MI-induced cardiac dysfunction^31^, ischemia‒reperfusion injury in murine cardiomyocytes^32,33^, atrial fibrillation-induced myocardial fibrosis^34^, and pressure overload-induced cardiomyocyte hypertrophy^35^. In the present study, cardiac miR-30a-5p expression was downregulated by natural senescence or D-gal-induced aging, suggesting the involvement of miR-30a-5p in regulating cardiac aging. Interestingly, miR-30a-5p deficiency aggravated cardiac senescence. Moreover, cardiac-specific overexpression of miR-30a-5p had a protective effect on D-gal-induced cardiac dysfunction and senescence. The p53 and p21 pathways are pivotal for establishing senescence after their activation by stress^36^. In our study, p53 and p21 were regulated by the deficiency or overexpression of miR-30a-5p in senescent hearts or cardiomyocytes.

Cellular senescence contributes to a decline in tissue regenerative capacity and function, inducing inflammation and pathological remodeling in aged organs^9^. Cell cycle arrest is not a hallmark of cardiomyocyte senescence because cardiomyocytes are terminally differentiated cells^37^. Thus, the senescence of cardiomyocytes is not as easy to define as that of proliferative or stem cells. Cardiomyocyte senescence is generally characterized by a decline in various functions, including the DNA damage response, endoplasmic reticulum stress, mitochondrial dysfunction, contractile dysfunction, and hypertrophic growth^38^. While different mechanisms can induce senescence in cardiomyocytes, stressors predominate in inducing disease phenotypes in cardiomyocytes^39^. In the present study, after D-gal treatment-induced or natural aging, miR-30a-5p deficiency aggravated heart dysfunction, increased the number of SA-β-gal-positive cells, and increased p53 and p21 levels, indicating the essential role of miR-30a-5p in the regulation of heart aging. Telomere shortening occurs naturally with aging^14^. Critically short telomeres are associated with cellular senescence and CVD^40–42^. Cardiomyocyte-specific telomere attrition is a characteristic of HF in humans and is associated with decreased contractile ability, hypertrophy, and senescence^43,44^. During heart aging, senescent cardiomyocytes exhibit impaired shortening, increased pacing frequency, and contractile and metabolic dysfunction^45^. Unveiling the molecular mechanisms responsible for preserving telomeres in cardiomyocytes may provide valuable insights into antagonizing cellular aging and maintaining cardiac function. In this study, telomere length was significantly decreased in senescent miR-30a-5p-deficient hearts and aged NRCMs. Additionally, the overexpression of miR-30a-5p in aging hearts and senescent cardiomyocytes increased telomere length both in vivo and in vitro, indicating that miR-30a-5p may be a critical molecule for preserving telomeres in cardiomyocytes. Moreover, the mechanism underlying the regulation of telomere length by miR-30a-5p may be attributed to the telomere enzymes TERT and TERF2.

In mice, TP53INP1 is encoded by *Tp53inp1*^46^. *Tp53inp1* is a target gene of p53, and conversely, the TP53INP1 protein, which is localized in the nucleus, positively modulates p53 transcriptional activity, mediating antioxidant-associated tumor suppressor functions^28^. TP53INP1 is involved mainly in mediating apoptotic cell death via the activation of p53 signaling^47^. In the present study, we revealed that *Tp53inp1* is a critical target of miR-30a-5p. In addition, TP53INP1 protein expression was decreased by miR-30a-5p overexpression and was increased by its inhibitor. Furthermore, we found that miR-30a-5p deficiency facilitated TP53INP1 translocation from the cytoplasm to the nucleus and the interaction between TP53INP1 and p53. Our data also revealed that SUMOylation of TP53INP1 was promoted by miR-30a-5p deletion via regulation of the SUMOylation-related enzyme SENP1.

Overall, our findings demonstrate a novel mechanism by which the miR-30a-5p-mediated p53–TP53INP1 signaling pathway contributes to cardiomyocyte senescence, telomere maintenance, and myocardial aging, which is consistent with the cumulative evidence regarding the regulation of CVD phenotypes by miR-30a-5p. These findings may provide valuable information for the development of novel therapeutic strategies for HF and various other age-related disorders. However, our data have several limitations, such as the expression pattern of miR-30a-5p in the young and old hearts of humans, which needs to be validated, and the detailed mechanism by which miR-30a-5p regulates TP53INP1 SUMOylation should be further explored.

## Supplementary information

Supplementary Information

Original western blotting images

## Data Availability

All data supporting the findings of this study are available in the article and Supplementary Material files. All original data for this study can be obtained from the corresponding author.
