# Supplementary material for: SUMOylation of TP53INP1 is involved in miR-30a-5p-regulated heart senescence
Source: Exp Mol Med. 2024 Nov 7;56(11):2519–34. doi: 10.1038/s12276-024-01347-3 (PMC11612193; doi:10.1038/s12276-024-01347-3)
Supplement: Supplementary file 1 — Supplementary Information [file 12276_2024_1347_MOESM1_ESM.pdf]

Supplemental Material

SUMOylation of TP53INP1 Is Involved in miR-30a-5p-Regulated Heart Senescence

Yi-Xiang Hong[1], Chan Wu[1], Jing-Zhou Li[1], Fei Song[1], Yu Hu[1], Yue Han[1], Yi-Jie Mao[1], Wei-Yin Wu[1,2], Yan Wang[1,2*], and Gang Li[1,2*]

1. Xiamen Cardiovascular Hospital of Xiamen University, School of Medicine, Xiamen University, Xiamen, Fujian (361000), China

2. Xiamen Key Laboratory of Cardiovascular Diseases, Xiamen, Fujian (361000), China

Running title: TP53INP1 in miR-30a-5p-Regulated Heart Aging

***Correspondence to:**

Gang Li, Xiamen Cardiovascular Hospital of Xiamen University, School of Medicine, Xiamen University, Xiamen, Fujian (361000), China. Email address: ligang@xmu.edu.cn

Yan Wang, Xiamen Cardiovascular Hospital of Xiamen University, School of Medicine, Xiamen University, Xiamen, Fujian (361000), China. Email address: wy@medmail.com.cn

SUPPLEMENTAL FIGURES AND FIGURE LEGENDS

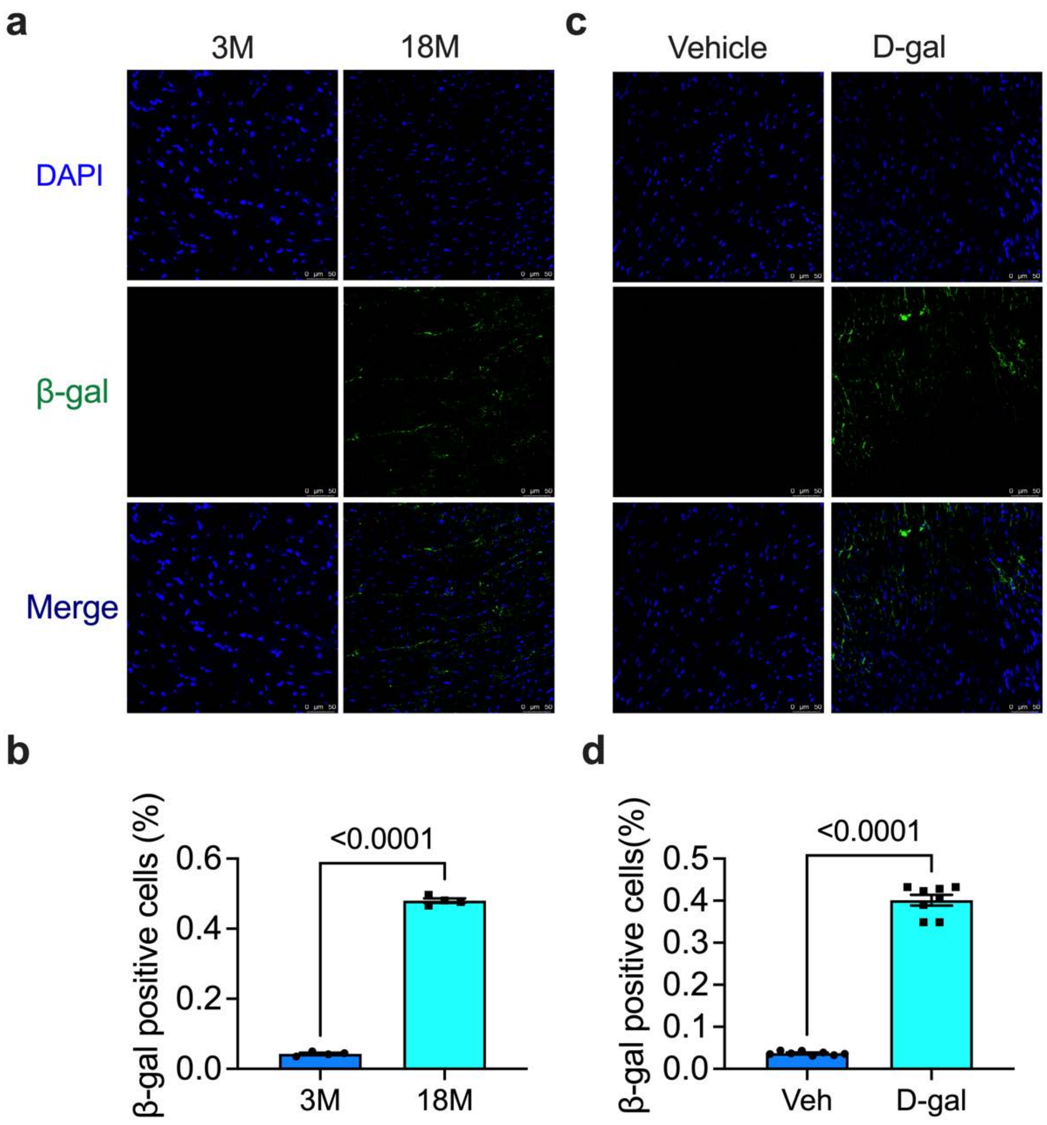

Supplementary Fig. 1. Natural- or D-gal-induced senescence promoted β-gal expression in the heart. a, Representative immunostaining images of β-gal positive cells in the hearts of 3- and 18-month-old mice (scale bars=50 μm). **b**, Statistical analysis of β-gal positive cells in the hearts of 3- and 18-month-old mice (n=6 in each group). **c**, Representative immunostaining images of β-gal positive cells in the hearts of vehicle- and D-gal-treated mice (scale bars=50 μm). **d**, Statistical analysis of β-gal positive cells in the hearts of 3 months and 18 months mice (n=8 in each group).

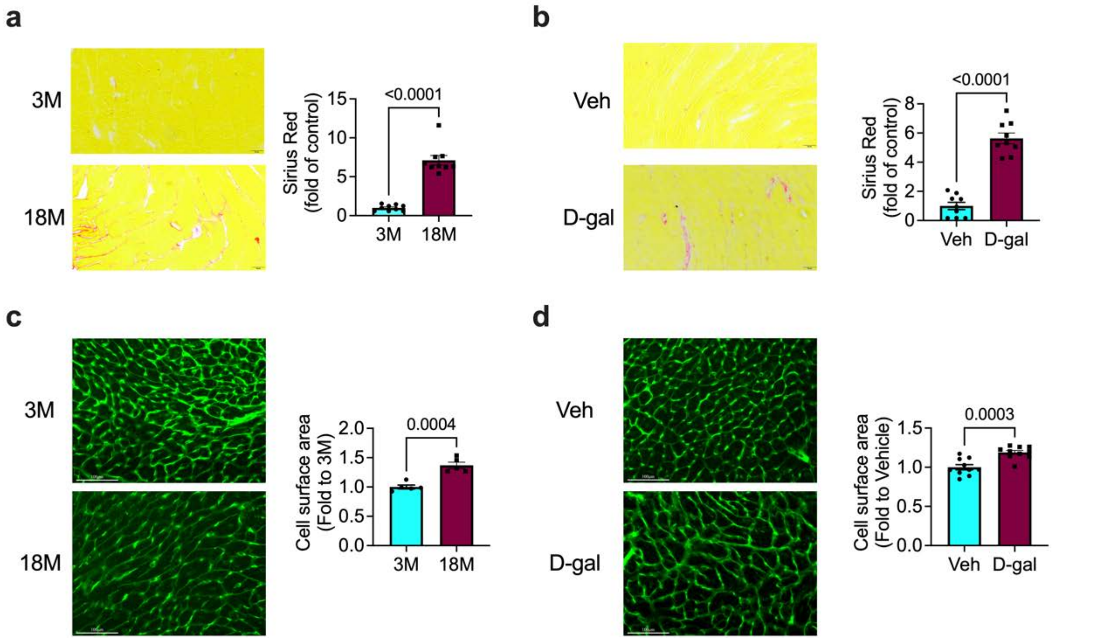

Supplementary Fig. 2. Heart aging promoted cardiac fibrosis and hypertrophy.

a, Representative images and statistical analysis of Picro Sirius red-stained heart tissues of 3- and 18-month-old mice (n=9 in each group, scale bars=50 μm). **b**, Representative images and statistical analysis of Picro-Sirius red stained heart tissues of vehicle (Veh) and D-gal-treated mice (n=9 in each group, scale bars=50 μm). **c**, Representative images and statistical analysis of Wheat Germ Agglutinin (WGA)-stained heart tissues of 3- or 18-month-old mice (n=5 in each group, scale bars=100 μm). **d**, Representative images and statistical analysis of WGA-stained heart tissues of vehicle (Veh)- and D-gal-treated mice (n=10 in each group, scale bars=100 μm).

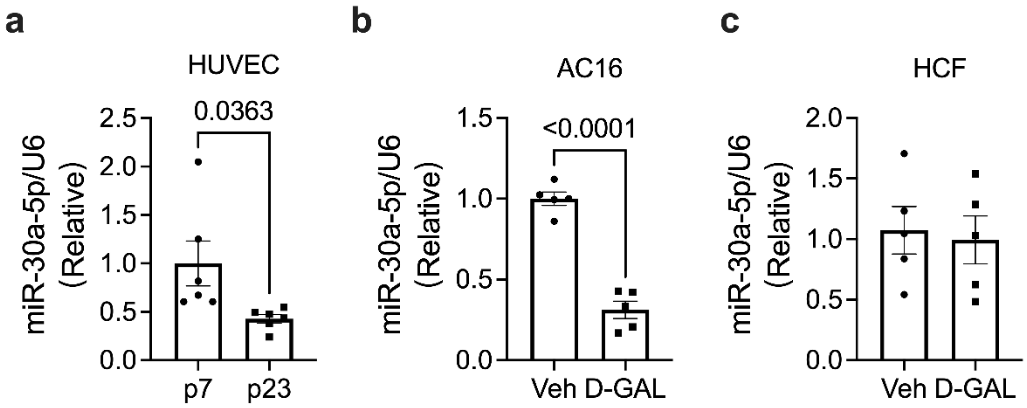

Supplementary Fig. 3. Expression of miR-30a-5p in human aging cells.

a, qRT-PCR analysis of the expression of miR-30a-5p in the human umbilical vein endothelial cells (HUVECs) at passage 7 and 23 (n=6 in both groups). **b**, qRT-PCR analysis of the expression of miR-30a-5p in the AC16 human cardiomyocytes (AC16) treated with vehicle (Veh) or D-gal (n=5 in both groups). **c**, qRT-PCR analysis of the miR-30a-5p expression in the human cardiac fibroblasts (HCFs) treated with vehicle or D-gal (n=5 in each group).

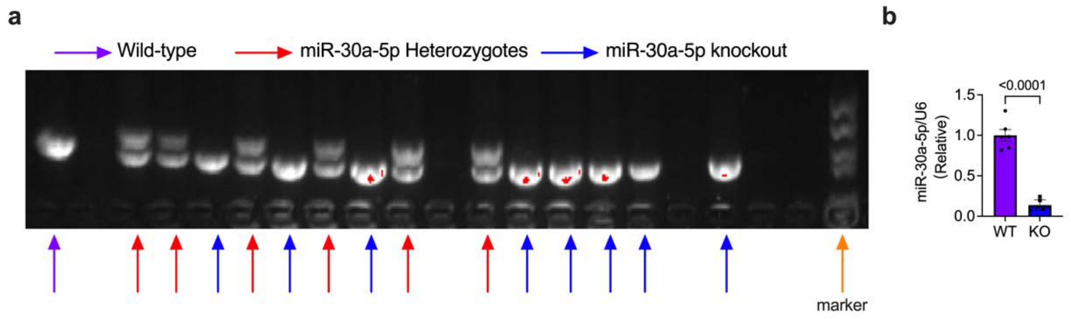

Supplementary Fig. 4. Efficiency of miR-30a-5p knockout in mice.

a, Representative genotyping of miR-30a-5p knockout mice. Purple arrows indicate wild-type (WT) mice, red arrows heterozygous (miR-30a-5p$^{+/-}$) mice, and blue arrows miR-30a-5p knockout (miR-30a-5p$^{-/-}$, KO) mice. **b**, qRT-PCR analysis of the miR-30a-5p expression in the hearts of WT and KO mice (n=6 in each group).

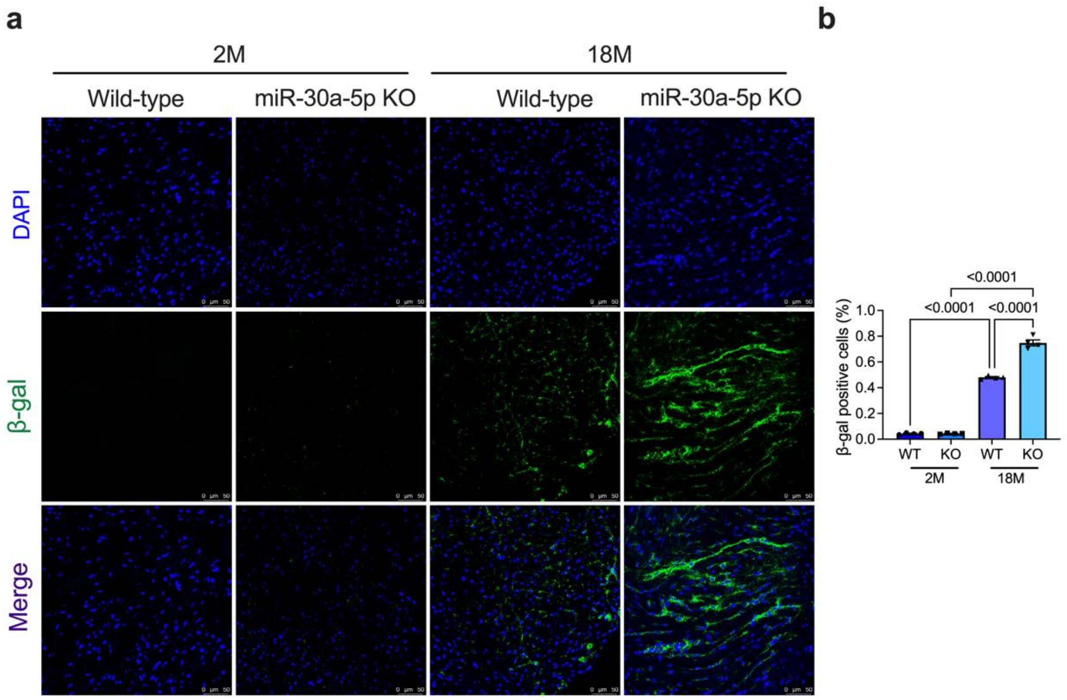

Supplementary Fig. 5. knockout of miR-30a-5p promoted β-gal expression in the aging heart.

a, Representative immunostaining images of β-gal positive cells in 2- or 18-month-old wild-type (WT) or miR-30a-5p knockout (KO) mice (scale bars=50 μm). **b**, Statistical analysis of β-gal positive cardiomyocytes in 2- or 18-month-old WT or KO mice (n=4 in each group).

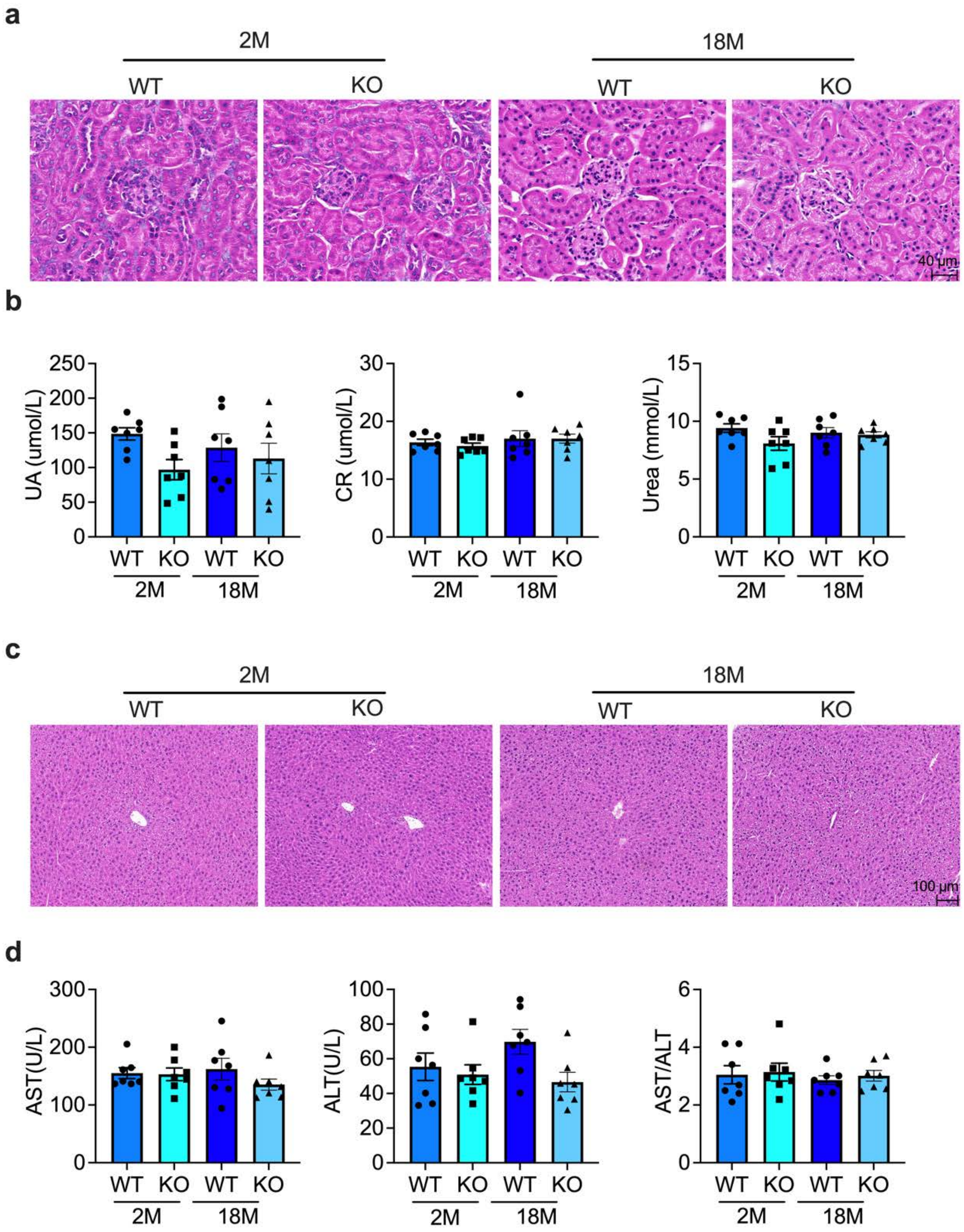

Supplementary Fig. 6. KO have no impact on aging kidney and liver.

a, H&E staining of the kidney of 2- or 18-month-old wild-type (WT) or miR-30a-5p knockout (KO) mice (scale bars=40 μm). **b**, Statistical analysis of uric acid (UA), creatinine (CR), and Urea level in the blood of 2- or 18 months-old WT or KO mice (n=7 in each group). **c**, H&E staining of the liver of 2- or 18-month-old WT or KO mice

(scale bars=100 μm). **d**, Statistical analysis of aspartate transaminase (AST), alanine aminotransferase (ALT), and the ratio of AST/ALT in the blood of 2- or 18-month-old WT or KO mice (n=7 in each group).

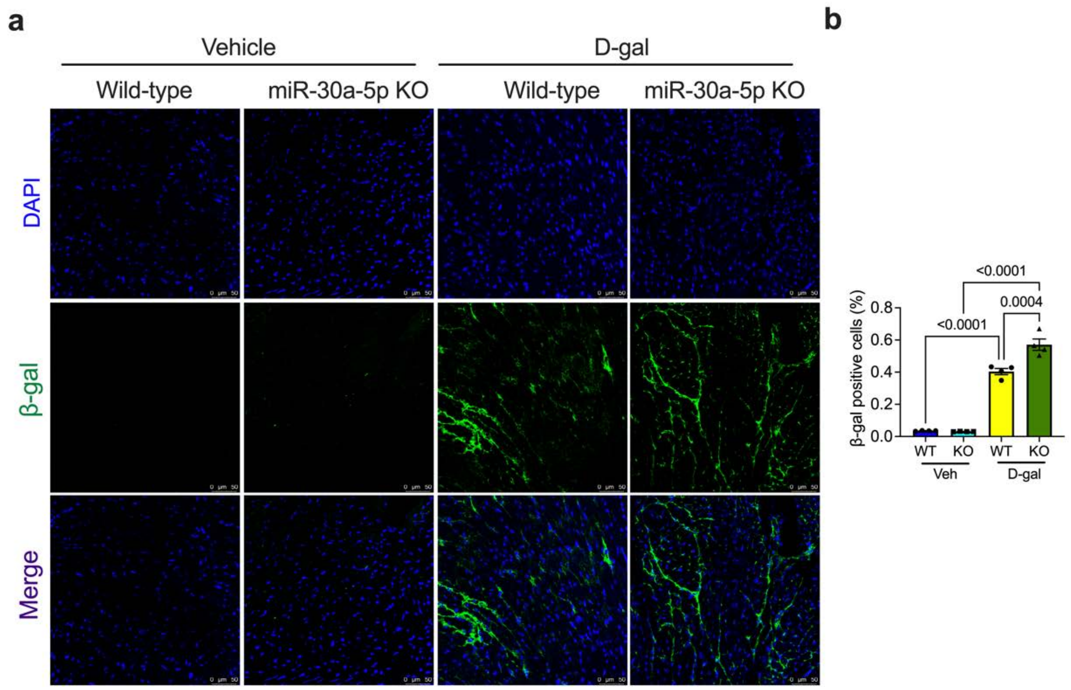

Supplementary Fig. 7. KO increased β-gal level in the D-gal-induced aging heart.

a, Representative immunostaining images of β-gal positive cells in vehicle- or D-gal-treated wild-type or miR-30a-5p KO mice (scale bars=50 μm). **b**, Statistical analysis of β-gal positive cells in vehicle (Veh)- or D-gal-treated wild-type (WT) or miR-30a-5p KO (KO) mice (n=4 in each group).

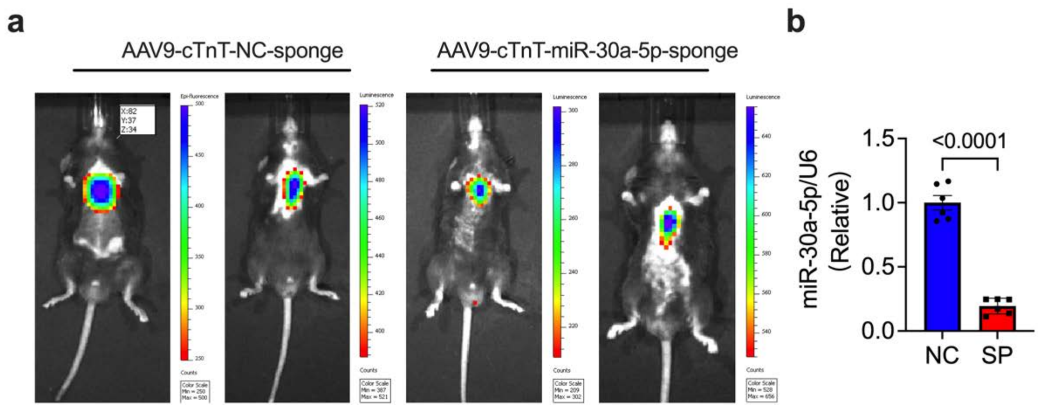

Supplementary Fig. 8. Efficiency of cardiac-specific knockdown of miR-30a-5p in wild-type mice.

a, Representative luciferase images (Caliper IVIS Lumina II *in vivo* imaging system) showing wild-type mice transfected with AAV9-cTnT-NC-sponge (NC) or AAV9-cTnT-miR-30a-5p-sponge (SP). **b**, qRT-PCR analysis of the miR-30a-5p expression in the hearts of wild-type mice transfected with NC or SP.

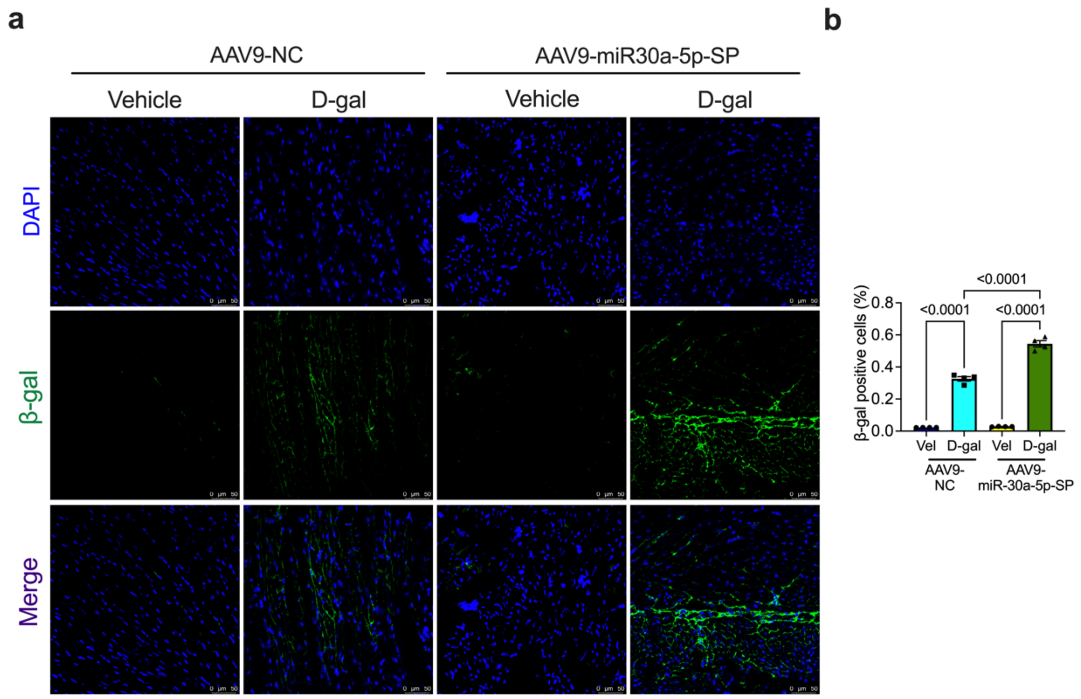

Supplementary Fig. 9. Cardiac specific knockdown of miR-30a-5p increased β-gal-positive cells in the aging heart.

a, Representative immunostaining images of β-gal-positive cells in AAV9-control (AAV9-NC) or AAV9-miR-30a-5p sponge (AAV9-miR-30a-5p-SP)-infected wild-type mice induced with vehicle and D-gal (scale bars=50 μm). **b**, Statistical analysis of β-gal-positive cells in the heart of AAV9-control (AAV9-NC) or AAV9-miR-30a-5p sponge (AAV9-miR-30a-5p-SP)-infected wild-type mice induced with vehicle and D-gal (n=4 in each group).

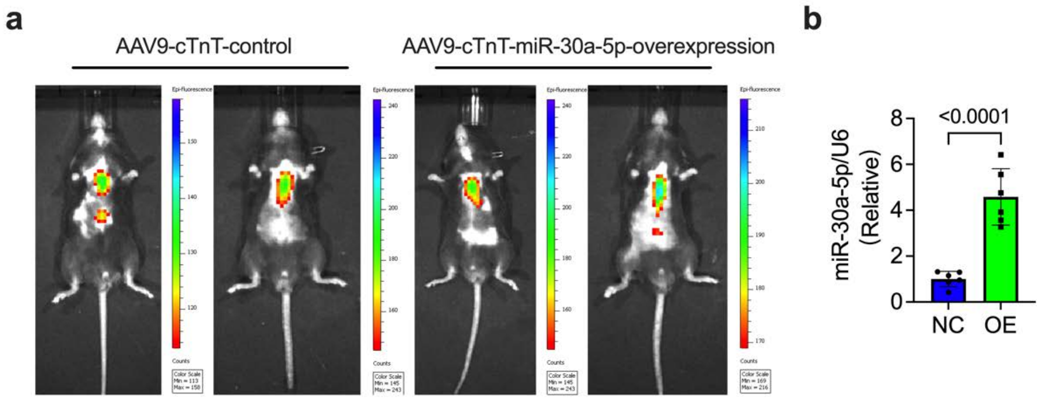

Supplementary Fig. 10. Efficiency of cardiac-specific overexpression of miR-30a-5p in wild-type (WT) mice.

a, Representative luciferase images (Caliper IVIS Lumina II *in vivo* imaging system) of AAV9-cTNT-control (NC) or AAV9-cTnT-miR-30a-5p-overexpressing WT mice. **b**, qRT-PCR quantification of the miR-30a-5p expression in the heart of WT mice transfected with AAV9-cTnT-control (NC) or AAV9-cTnT-miR-30a-5p overexpression (OE) vectors.

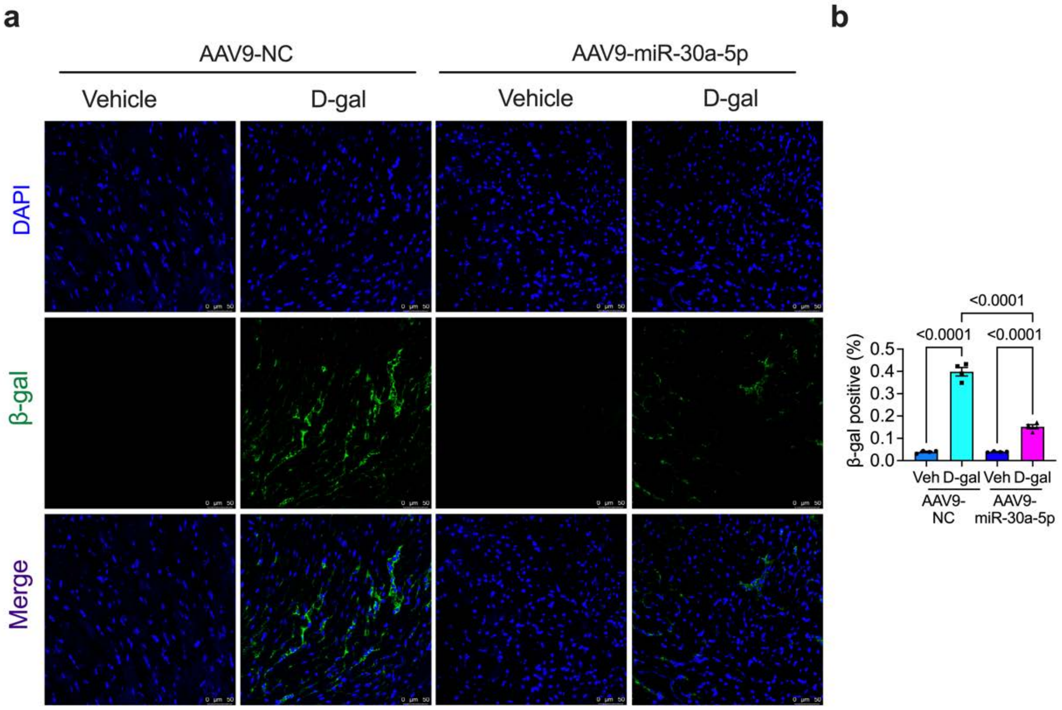

Supplementary Fig. 11. Overexpression of miR-30a-5p decreased β-gal positive cells in D-gal-induced aging hearts.

a, Representative immunostaining images of β-gal positive cardiomyocytes in AAV9-control (AAV9-NC) or AAV9-miR-30a-5p-infected wild-type mice treated with vehicle and D-gal (scale bars=50 μm). **b**, Statistical analysis of β-gal positive cells in the heart of AAV9-control (AAV9-NC) or AAV9-miR-30a-5p-infected wild-type mice treated with vehicle and D-gal (n=4 in each group).

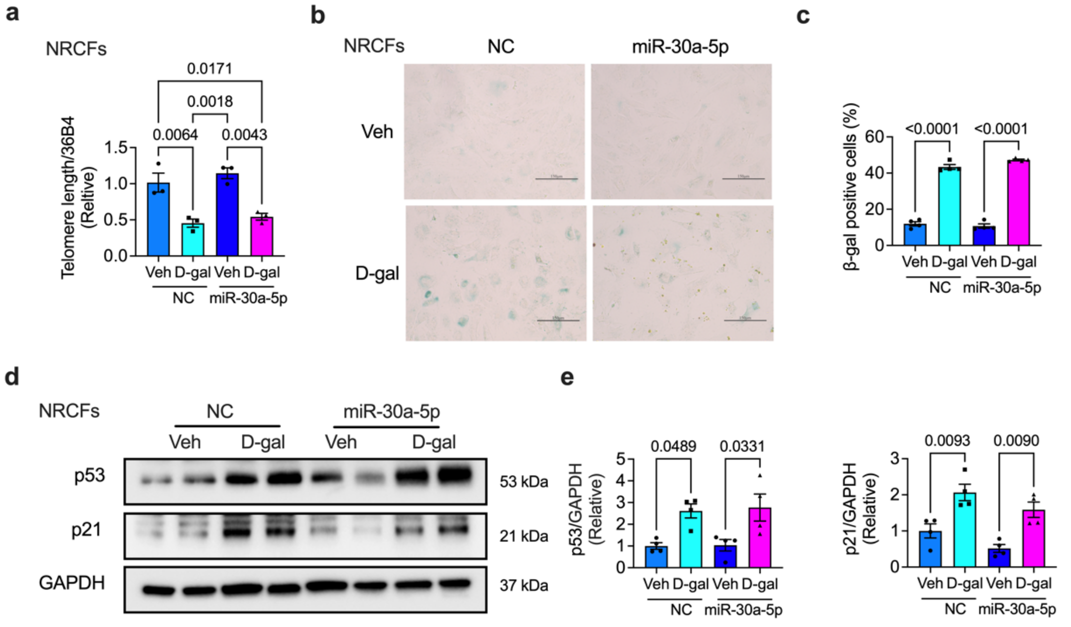

Supplementary Fig. 12. Overexpression of miR-30a-5p not alleviates D-gal-induced senescence in neonatal rat cardiac fibroblasts (NRCFs). a, qRT-PCR analysis of telomere length of NRCFs transfected with control mimic or miR-30a-5p mimic induced by with vehicle or D-gal (n=3 in each group). **b** and **c**, Representative SA-β-gal staining images **(b)** and analysis **(c)** of NRCFs treated with vehicle or D-gal transfected with control or miR-30a-5p mimic (n=4 in each group, scale bars=150 μm). **d** and **e**, Representative western blotting images **(d)** and analysis **(e)** of p53 and p21 expression in NRCFs treated with vehicle or D-gal transfected with control or miR-30a-5p mimics (n=4 in each group).

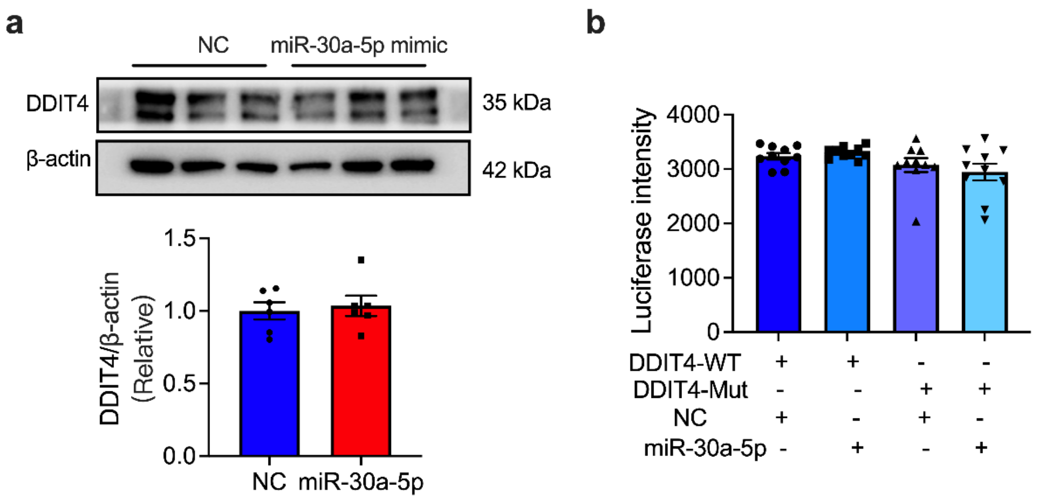

Supplementary Fig. 13. Overexpression of miR-30a-5p unaffected the levels and activity of DDIT4. a, Representative western blotting images and quantitative analysis of DDIT4 in NRCMs transfected with control (NC) or miR-30a-5p mimic (n=6 in each group). **b**, Luciferase activity assay of NRCMs co-transfected with NC or miR-30a-5p mimic and WT or mutant DDIT4 (n=10 in each group).

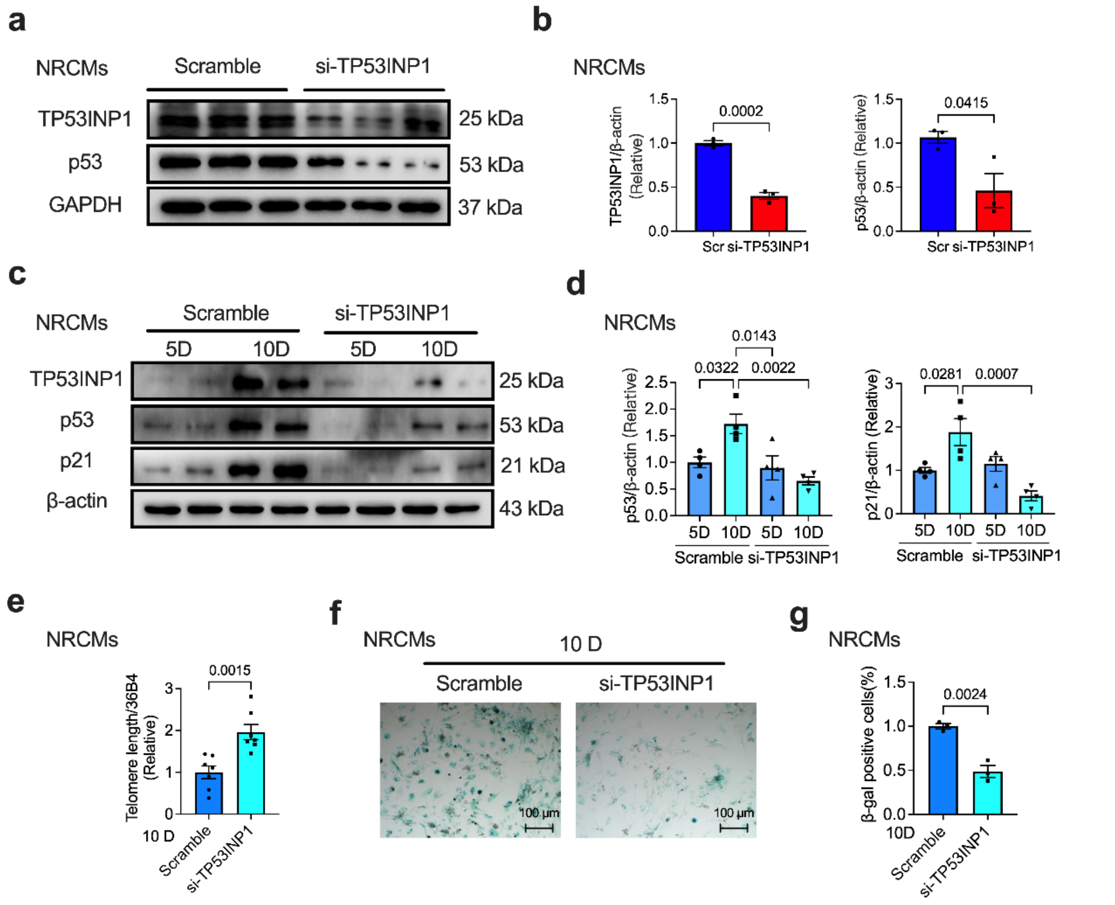

Supplementary Fig. 14. Knockdown of TP53INP1 alleviates natural aging-induced senescence of neonatal rat cardiomyocytes (NRCMs).

a and **b**, Representative western blotting images **(a)** and analysis **(b)** of TP53INP1 and p53 expression in NRCMs transfected with scramble siRNA (Scr) or *Tp53inp1* siRNA (si-TP53INP1) (n=3 in each group). **c** and **d**, Representative western blotting images **(c)** and analysis **(d)** of TP53INP1, p53, and p21 expression in 5- or 10-day-treated NRCMs transfected with scramble siRNA (Scramble) or *Tp53inp1* siRNA (si-TP53INP1) (n=4 in each group). **e**, qRT-PCR analysis of telomere length of 10-day-treated NRCMs transfected with scramble siRNA (Scramble) or *Tp53inp1* siRNA (si-TP53INP1) (n=6 in each group). **f** and **g**, Representative senescence-associated β-galactosidase (SA-β-gal) staining images **(f)** and analysis **(g)** of 10-day-treated NRCMs transfected with

scramble siRNA (Scramble) or *Tp53inp1* siRNA (si-TP53INP1) (n=3 in each group, scale bars=100 μm).

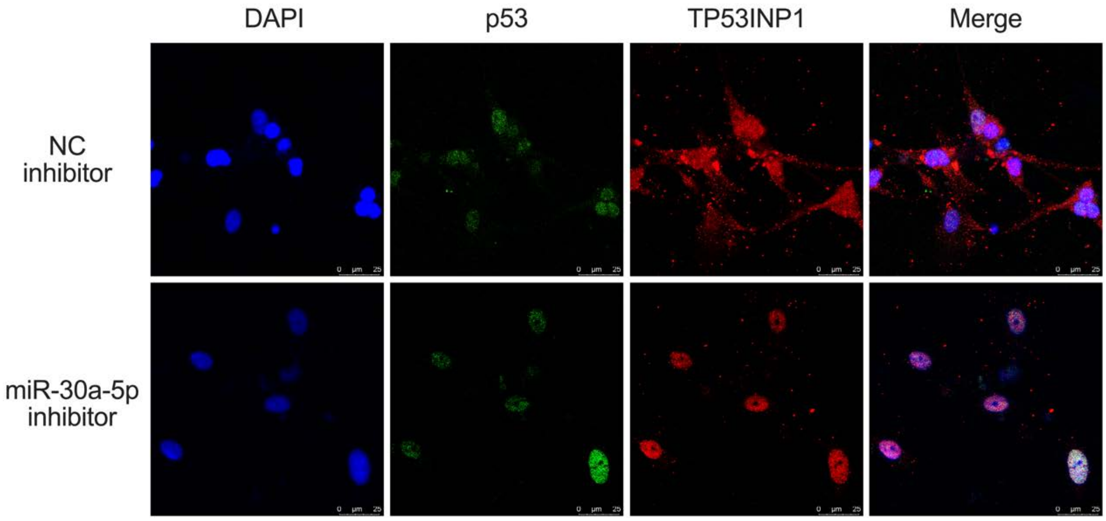

Supplementary Fig. 15. Immunofluorescence of p53 (green) and TP53INP1 (red) in NRCMs transfected with Negative control inhibitor or miR-30a-5p inhibitor (scale bars=25 μm).

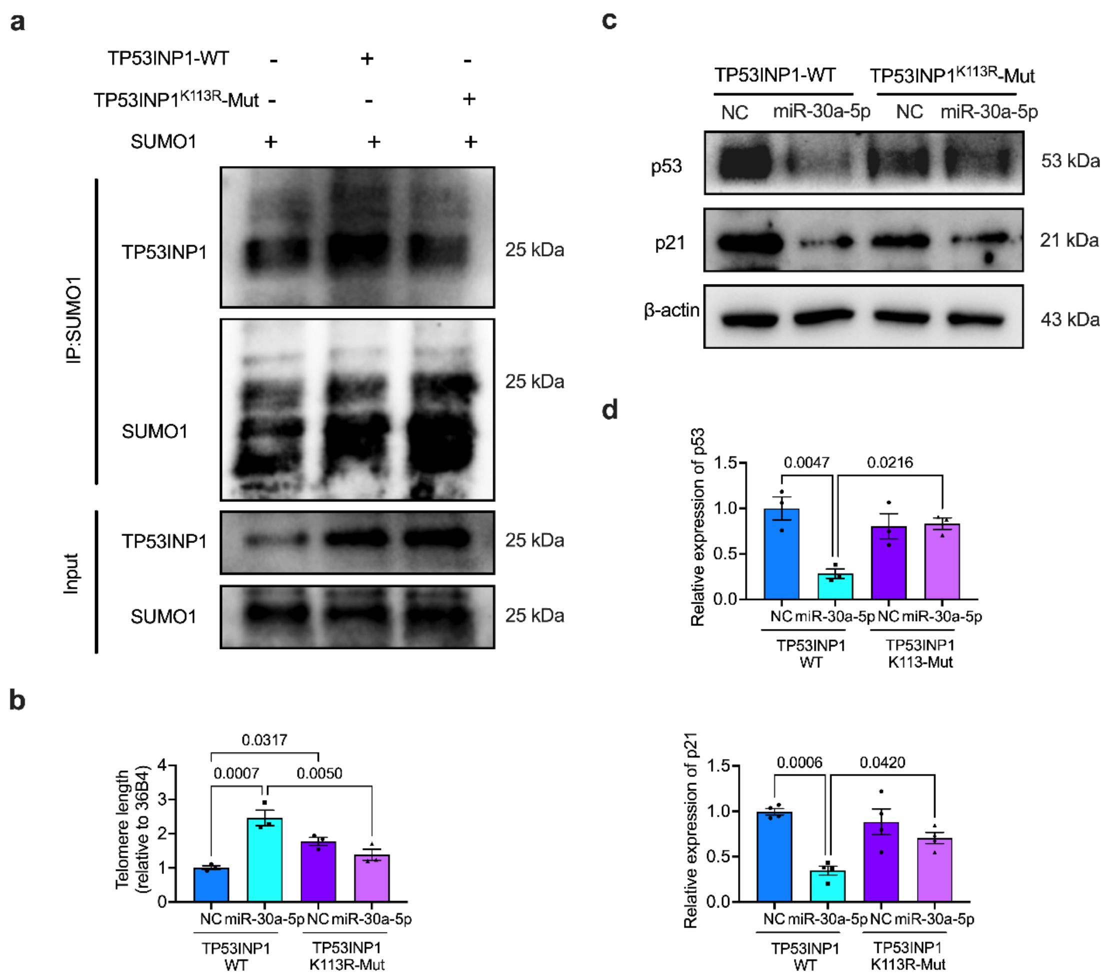

Supplementary Fig. 16. K113R SUMOylation of TP53INP1 participates in miR-30a-5p-regulated cardiomyocytes aging.

a, Immunoprecipitation and Western blot tested the SUMO1 level in AC16 human cardiomyocyte co-transfected with SUMO1 and TP53INP1 or TP53INP1 K113R mutant. **b,** qPCR analysis of human AC16 cardiomyocytes transfected with control or miR-30a-5p mimic and wild-type (WT) TP53INP1 or mutant K113R of TP53INP1 (n=3). **c** and **d**, Western blotting images **(c)** and analysis **(d)** of p53 (n=3) and p21 (n=4) protein levels in human AC16 cardiomyocytes transfected with control or miR-30a-5p mimic and wild-type (WT) TP53INP1 or mutant K113R of TP53INP1.

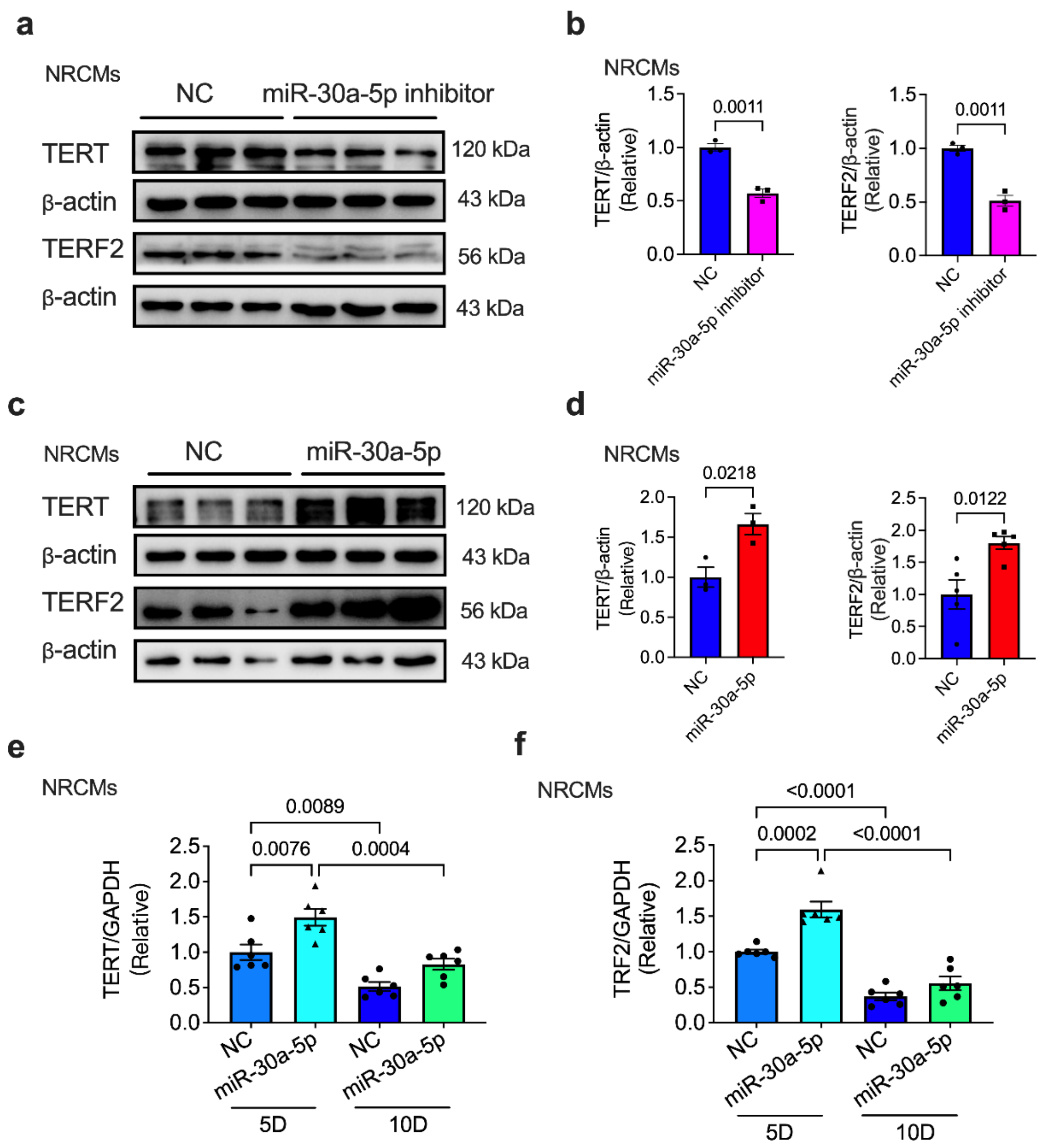

Supplementary Fig. 17. miR-30a-5p regulates TERT and TERF2 expression in naturally aged neonatal rat cardiomyocytes (NRCMs).

a and **b**, Western blotting images **(a)** and analysis **(b)** of TERT and TERF2 protein levels in NRCMs transfected with control (NC) or miR-30a-5p inhibitor (n=3 in each group). **c** and **d**, Western blotting images **(c)** and analysis **(d)** of TERT and TERF2 protein levels in NRCMs transfected with control (NC) or miR-30a-5p mimic (miR-30a-5p) (n=3 in each group). **e** and **f**, qRT-PCR analysis of *Tert* **(e)** and *Terf2* **(f)** mRNA levels of 5- or

10-day-treated NRCMs transfected with control (NC) or miR-30a-5p mimic (n=6 in each group).

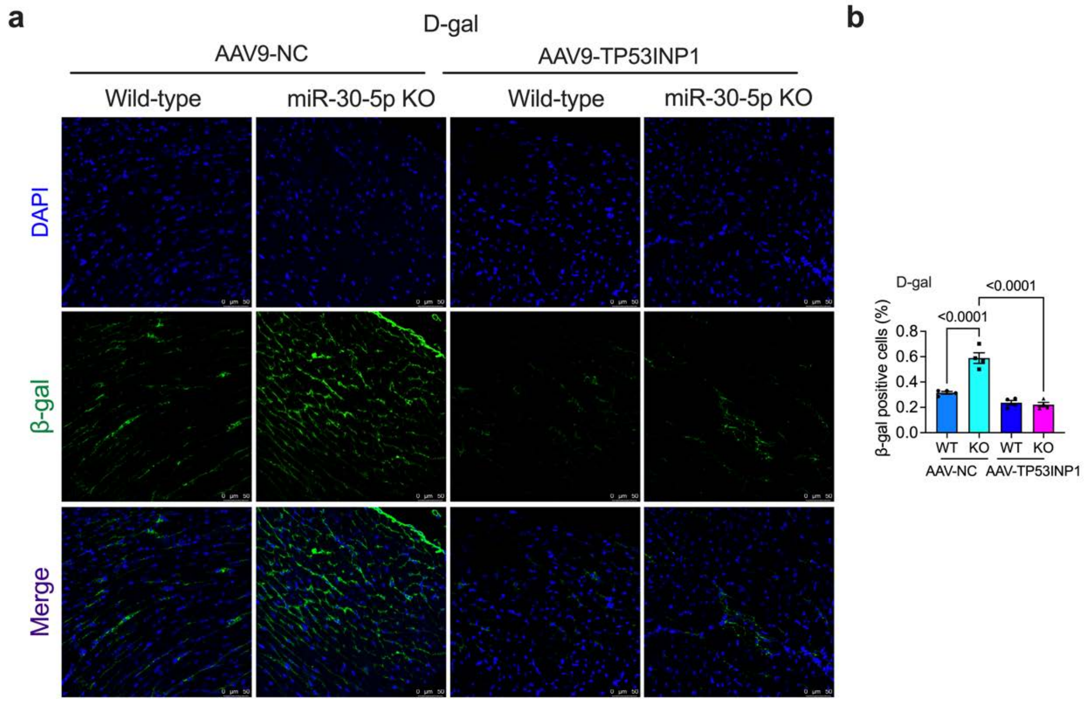

Supplementary Fig. 18. TP53INP1 silencing decreased β-gal-positive cells in D-gal-treated KO hearts.

a, Representative immunostaining images of β-gal-positive cardiomyocytes in AAV9-control (AAV9-NC) or AAV9-TP53INP1 RNAi (AAV9-TP53INP1)-infected wild-type (WT) or KO mice treated with D-gal (scale bars=50 μm). **b**, Statistical analysis of β-gal-positive cells in the heart of AAV9-control (AAV9-NC) or AAV9-TP53INP1 RNAi (AAV9-TP53INP1)-infected WT or KO mice treated with D-gal (n=4 in each group).
